# Roll‐To‐Roll Production of Smart Dressings for Wound Monitoring

**DOI:** 10.1002/adhm.202501998

**Published:** 2025-07-22

**Authors:** Ziheng Wang, Yujin Ahn, Semin Kwon, Tianhao Yu, Yumin Dai, Julia Walsh, Joo Hun Lee, Sang Mok Park, Seul Ah Lee, Murtuza Peerbhai, Chandan K. Sen, Hyowon Lee, Young L. Kim, Hyunjoon Kong, Chi Hwan Lee

**Affiliations:** ^1^ School of Mechanical Engineering Purdue University West Lafayette IN 47907 USA; ^2^ Department of Chemical and Biomolecular Engineering University of Illinois at Urbana−Champaign Urbana IL 61801 USA; ^3^ Weldon School of Biomedical Engineering Purdue University West Lafayette IN 47907 USA; ^4^ School of Materials Engineering Purdue University West Lafayette IN 47907 USA; ^5^ Monticello Podiatry, LLC, Monticello, IN, 47960, USA; ^6^ McGowan Institute for Regenerative Medicine University of Pittsburgh Pittsburgh PA USA; ^7^ Department of Surgery University of Pittsburgh Pittsburgh PA USA; ^8^ Department of Plastic Surgery University of Pittsburgh Pittsburgh PA USA; ^9^ Carl R. Woese Institute for Genomic Biology University of Illinois at Urbana−Champaign Urbana IL 61801 USA; ^10^ Elmore Family School of Electrical and Computer Engineering Purdue University West Lafayette IN 47907 USA

**Keywords:** colorimetric sensing, Roll‐to‐roll (R2R) manufacturing, smart wound dressings, wound healing assessment

## Abstract

Chronic wounds require real‐time monitoring to assess wound status and prevent complications. While colorimetric sensors provide a user‐friendly, equipment‐free approach with visible feedback, their clinical adoption is hindered by limited scalability, poor integration with commercial dressings, and susceptibility to interference from wound exudates—factors that have largely confined existing systems to laboratory research and prevented their practical use in clinical settings. Here, the first roll‐to‐roll production of smart dressings at a large scale and high throughput, with colorimetric sensors for real‐time and historical wound monitoring is presented. This method enables high‐throughput production, achieving over 10 impressions per minute on formats up to 50 cm × 50 cm, using biocompatible inks for precise multi‐layer printing. In situ testing on a 14‐day mouse wound model demonstrates their ability to detect early signs of infection, validating their functionality in physiologically relevant conditions. This study addresses the manufacturability gap in colorimetric wound sensing, providing a scalable, cost‐effective, and clinically translatable solution for both clinical and home use.

## Graphical abstract

This study presents a roll‐to‐roll manufactured smart wound dressing capable of visually monitoring key wound biomarkers, including pH, humidity, and temperature. By integrating colorimetric sensors into flexible dressing substrates, the device offers real‐time, equipment‐free insights for infection detection and wound management, representing a scalable solution for personalized healthcare.

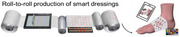

## 1 Introduction

Chronic wounds, defined as wounds that fail to heal within three months and often remain in a prolonged state of inflammation, present a significant burden to both patients and healthcare systems.^[^ ^1^, ^2^, ^3^ ^]^ In the United States alone, ≈6.5 million patients are affected by chronic wounds, with annual treatment costs exceeding $25 billion.^[^ ^4^, ^5^ ^]^ These wounds include venous ulcers, pressure ulcers, burn wounds, and diabetic foot ulcers (DFUs), all of which can lead to complications such as increased pain, persistent inflammation, infection, and tissue necrosis, hindering healing and worsening patient outcomes.^[^ ^3^, ^6^ ^]^ Among these complications, infection plays a major role in severe medical conditions, contributing significantly to diabetes‐related lower limb amputations globally each year and driving the high five‐year post‐amputation mortality rate.^[^ ^7^, ^8^, ^9^ ^]^

Current clinical methods for wound assessment primarily rely on visual evaluations by clinicians and bacterial cultures from swabs, a process with long turnaround times that often delays timely diagnosis.^[^ ^10^ ^]^ These challenges are even more pronounced in rural areas, where limited access to healthcare professionals and laboratory facilities makes timely wound care difficult.^[^ ^11^ ^]^ This underscores the need for real‐time, in situ wound monitoring systems that are both low‐cost and suitable for home use.^[^ ^12^, ^13^ ^]^ To address these issues, ongoing research has focused on developing smart wound dressings that incorporate wearable electronic or colorimetric sensors to monitor key wound biomarkers—such as pH, humidity, uric acid, glucose, temperature, and inflammatory factors—for early detection of complications.^[^ ^14^, ^15^, ^16^, ^17^, ^18^, ^19^, ^20^, ^21^, ^22^, ^23^ ^]^ Colorimetric sensors are particularly favorable in disposable healthcare devices because they offer visible, easy‐to‐interpret results without requiring complex electronics, power supplies, or wireless communication components.^[^ ^24^, ^25^, ^26^, ^27^ ^]^ Recent advancements in colorimetric sensing strategies have integrated color‐changing dyes, nanomaterials, and biomimetic elements into hydrogel patches, microfluidic devices, and functionalized textiles, improving chronic wound detection and reducing the effort involved in wound management.^[^ ^19^, ^28^, ^29^, ^30^ ^]^ Despite these advancements, many colorimetric wound sensors remain at the laboratory stage due to challenges in scalability and integration into large‐scale manufacturing workflows.^[^ ^31^, ^32^ ^]^ Furthermore, most existing systems are designed to capture only real‐time information, limiting their ability to provide historical biomarker data for comprehensive wound assessment. This underscores the need for a scalable, cost‐effective fabrication approach capable of producing wound dressings that support both real‐time and time‐stamped biomarker monitoring for comprehensive wound assessment.

Roll‐to‐roll (R2R) manufacturing has gained attention as a scalable, high‐throughput approach for fabricating flexible electronics and biosensors on continuous substrates such as plastic films, paper, or textiles.^[^ ^33^, ^34^ ^]^ It has been widely applied in the development of flexible solar cells, organic light‐emitting diodes, and electronic displays, where large‐area coverage and mechanical compliance are essential.^[^ ^35^, ^36^, ^37^ ^]^ In biomedical applications, R2R printing has been used for wearable electrochemical sensors targeting sweat biomarkers such as pH, glucose, and electrolytes, while R2R hot embossing enables rapid fabrication of microfluidic channels for disposable diagnostics.^[^ ^38^, ^39^ ^]^ However, prior R2R‐based healthcare sensors require external power, signal processing, or post‐fabrication assembly, which can limit scalability and ease of use. Notably, no prior studies have reported the integration of colorimetric biosensors into wound dressings using R2R techniques. This gap highlights a compelling opportunity to leverage R2R manufacturing for colorimetric wound monitoring, enabling the scalable production of smart, low‐cost, and clinically deployable dressings.

Here, we present the first R2R production of smart dressings at a large scale and high throughput, with colorimetric sensors for real‐time and historical wound monitoring. The R2R process allows for the production of over 10 impressions per minute on formats as large as 50 cm × 50 cm, with a precise resolution of 0.4 mm using biocompatible inks. This system supports multi‐layer printing with precise registration and inline curing, allowing modular deposition of independently formulated inks onto textile‐type dressings and enabling functional expansion. We developed four colorimetric inks to detect pH, temperature, and humidity, each exhibiting reversible or irreversible color changes to provide both real‐time feedback (pH, temperature) and historical records of wound conditions (pH, humidity). The inks were optimized for R2R screen printing, supporting over 100 continuous imprints across 60 meters with consistent quality, demonstrating the feasibility of scalable, high‐throughput manufacturing. To enhance usability, we incorporated an anti‐fouling layer to minimize blood absorption and ensure clear visibility of color changes.^[^ ^40^, ^41^ ^]^ To ensure consistent readouts across imaging conditions, we implemented a smartphone‐based color regression model calibrated using a built‐in reference color checker placed adjacent to the sensors, enabling accurate and reproducible quantification of sensor responses across users, environments, and imaging devices. A 14‐day mouse model study demonstrated the dressing's effectiveness, with infected wounds showing elevated pH and temperature levels and delayed healing compared to non‐infected wounds. By providing real‐time data on key wound parameters, the dressing assists physicians in staging wounds and determining appropriate treatment strategies. This platform offers a cost‐effective and accessible solution suitable for both clinical and at‐home wound management.

## 2 Results

### 2.1 Design and R2R Production of the Smart Wound Dressing

**Figure 1:**
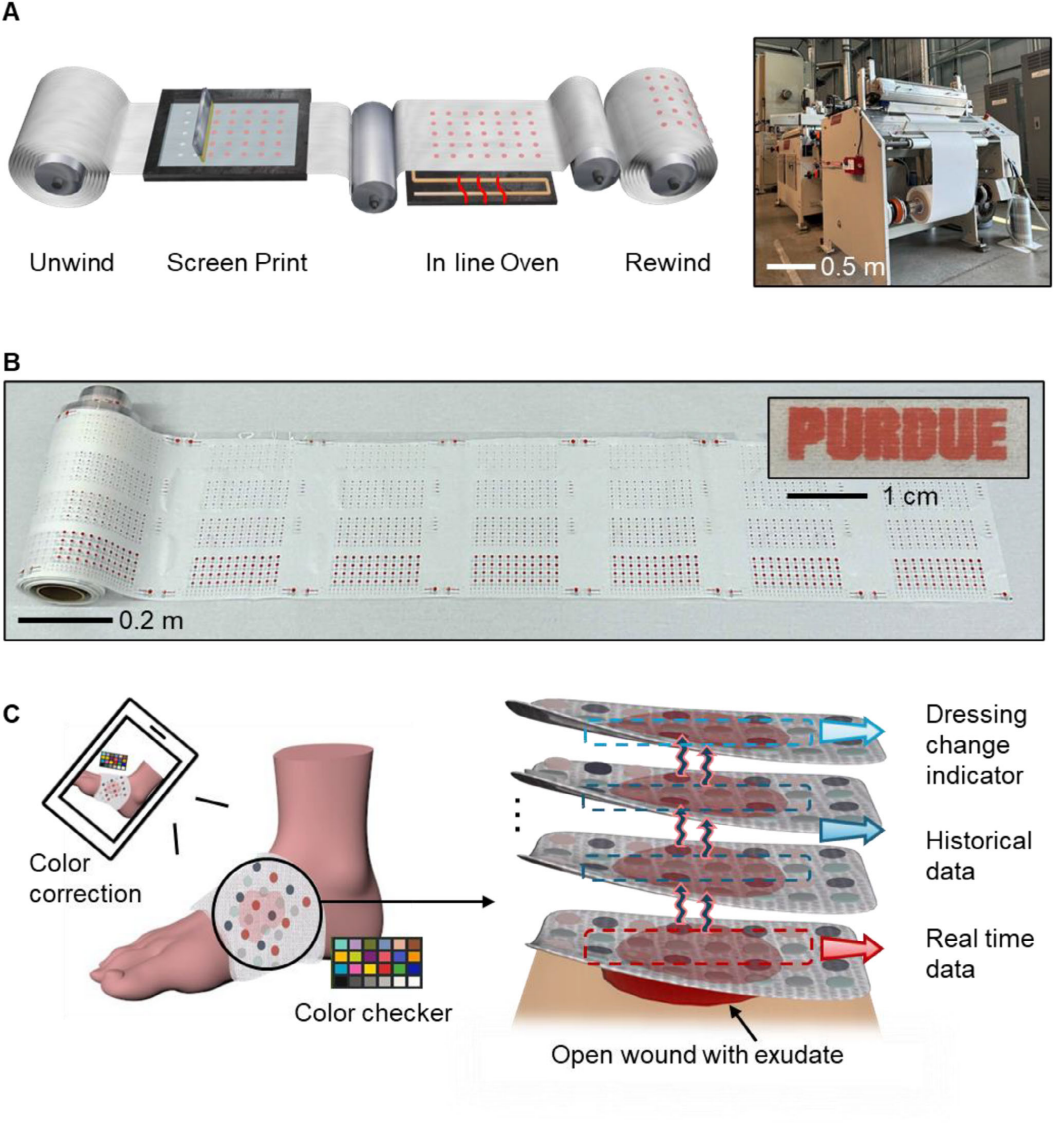
Schematics and smart wound dressing configuration. A) Schematic of the roll‐to‐roll printing process, with inset showing the roll‐to‐roll printing machine. B) Smart dressing roll printed on nonwoven fabric, with a patterned Purdue logo demonstrating customizable design. C) Illustration of the wound monitor and functional design of the smart wound dressing in the layered structure.

**Figure** **1**A illustrates a schematic of the R2R production process for the smart wound dressing using the Kinzel R2R machine. This machine, equipped with a vacuum conveyor belt, supports fully automated, continuous, and precise in‐line operations, enabling rapid, high‐throughput production—achieving over 10 impressions per minute, with each impression covering an area of 50cm × 50 cm, and extending up to 100 m. The fabrication process begins with unwinding the dressing roll, followed by precise alignment and screen printing of each sensor to form the sensing matrix. An antifouling layer is then applied, and the sensing inks are cured in an in‐line oven. Finally, the roll is rewound to produce a complete, ready‐to‐use dressing (Movie S1, Supporting Information). The sequential screen‐printing process integrates four biocompatible colorimetric inks, as model systems, each designed for specific sensing functions: 1) a reversible pH sensor (R. Ph), 2) an irreversible pH sensor (IR. Ph), 3) a reversible temperature (Temp.) sensor, and 4) an irreversible humidity sensor (Figure S1, Supporting Information). An antifouling layer is applied as the final step to enhance the longevity of the sensors. Each ink is specially formulated for screen printing, incorporating distinct indicators within a chitosan or polydimethylsiloxane (PDMS) matrix. To ensure consistent viscosity and high‐quality print definition, thickeners and plasticizers are added to optimize ink performance. Details of the ink formulation are provided in the Experimental Section. Figure S2A (Supporting Information) shows the viscosity profiles of the prepared inks, demonstrating precise screen‐printing capabilities with feature resolution down to 0.4 mm on a nonwoven substrate (Figure S2B, Supporting Information). The slightly reduced edge sharpness in the humidity and temperature sensors results from the shear‐thinning behavior and low surface tension of their PDMS‐based inks, which tend to spread slightly on the porous textile substrate before curing.

Figure 1B shows a photograph of the R2R‐fabricated smart wound dressing roll on a nonwoven substrate, with an inset highlighting the patterned Purdue logo printed using the developed reversible pH‐sensitive ink. Figure S3 (Supporting Information) further illustrates the patterning capabilities of this fabrication method across all sensing inks, along with the corresponding color changes under various conditions. A larger‐scale image of the smart dressing roll is provided in Figure S4 (Supporting Information). The high‐throughput nature of this fabrication process makes these sensors ideal as cost‐effective, disposable wound monitoring strips suitable for home‐based medical screening. Based on the retail purchasing prices of the ink components, the cost per imprint and per sensing unit of the smart wound dressing was ≈$1.59 and $0.01, respectively, as detailed in Table S1 (Supporting Information). Moreover, the fabrication method is specifically optimized for textile‐based dressings and is compatible with gauze, cotton, and nonwoven substrates commonly used in medical and personal care applications. Figures S5–S7 (Supporting Information) demonstrate color maps showing transitions for pH (4 to 10), temperature (30 to 40 °C), and moisture levels (dry to wet) across dressing substrates like cotton, gauze, and nonwoven materials. These results highlight the adaptability of the R2R screen‐printing process for diverse dressings, integrating traditional wound care with point‐of‐care colorimetric sensors for affordable, user‐friendly monitoring while maintaining a moist, hygienic wound environment.

Figure 1C illustrates the application of the smart dressing to a chronic wound with high exudate output, such as a DFU, demonstrating its adaptability in clinical settings. While the figure highlights a wrap‐around format suited for exudate‐rich wounds, the dressing can be easily cut and configured into various shapes and sizes (Figure S8, Supporting Information) to accommodate different wound types, anatomical locations, and body geometries. Depending on clinical needs, it can be applied as a single‐layer bandage for minor injuries, a patch for burns, or a multi‐layer wrap for chronic wounds (Movie S2, Supporting Information), offering the same usability as conventional gauze while enabling biomarker monitoring.

Designed for real‐time and retrospective colorimetric monitoring, sensor color changes are easily visible to the naked eye or can be precisely analyzed with a smartphone camera using a color checker for enhanced accuracy. The cloth dressing substrate supports natural skin functions, including oxygen and moisture exchange, while the top antifouling layer prevents blood clogging and maintains sensor visibility for reliable image capture and analysis. As the smart dressing absorbs wound exudate, each sensor changes color proportionally to the analyte concentration. The skin‐contact layer provides real‐time pH and temperature readings for both the wound and the surrounding healthy skin, serving as reference points. As wound exudate diffuses through the dressing, the middle layers capture peak pH and humidity levels over time, with sensor colors that can be inspected by gently lifting the dressing edge. The outermost layer functions as a dressing change indicator; when the humidity sensor changes from blue to red, signaling full saturation, it prompts the user to replace the dressing to maintain optimal wound hygiene.

The biomarker measurements enabled by the smart dressing provide critical medical benefits for managing chronic wounds. Table S2 (Supporting Information) highlights the clinical relevance of these biomarkers. Monitoring pH is particularly useful for early infection detection, as a shift from a neutral to an alkaline environment indicates bacterial proliferation and delayed healing.^[^ ^28^, ^42^, ^43^, ^44^ ^]^ The temperature sensor helps determine whether the wound is at the optimal healing temperature of 98.6 °F (37 °C), as maintaining a warm environment is crucial for wound healing. Reduced temperatures can inhibit cell migration and proliferation, often seen in cases of compromised blood flow, such as diabetic complications.^[^ ^45^ ^]^ Conversely, elevated temperatures may indicate infection, as excessive heat can negatively impact cytokines and growth factors, disrupting the healing process. The humidity sensor ensures optimal moisture levels for wound healing, balancing excessive moisture—which may encourage bacterial growth—and dryness, which can impede cellular migration and tissue regeneration.^[^ ^46^, ^47^ ^]^ However, since humidity levels vary depending on the type of wound dressing used,^[^ ^48^ ^]^ this measurement primarily serves to validate exudate management and confirm sensor activation in wounds with sufficient exudate. The smart dressing is particularly suited for exudative wounds, where it can assist in monitoring infection and provide timely cues for transitioning to a plain or moisturizing dressing as needed, enhancing its potential commercial viability for specialized wound care applications. Any alkaline pH or elevated temperature could suggest inflammation, while consistently alkaline pH, elevated temperature, or abnormal moisture levels indicate impaired wound healing. Collectively, these biomarker measurements enable clinicians and patients to monitor wound status more effectively, facilitating timely interventions, personalized care, and improved healing outcomes while reducing the risk of complications, including amputation.

### 2.2 Colorimetric Sensor Development and Calibration

**Figure 2:**
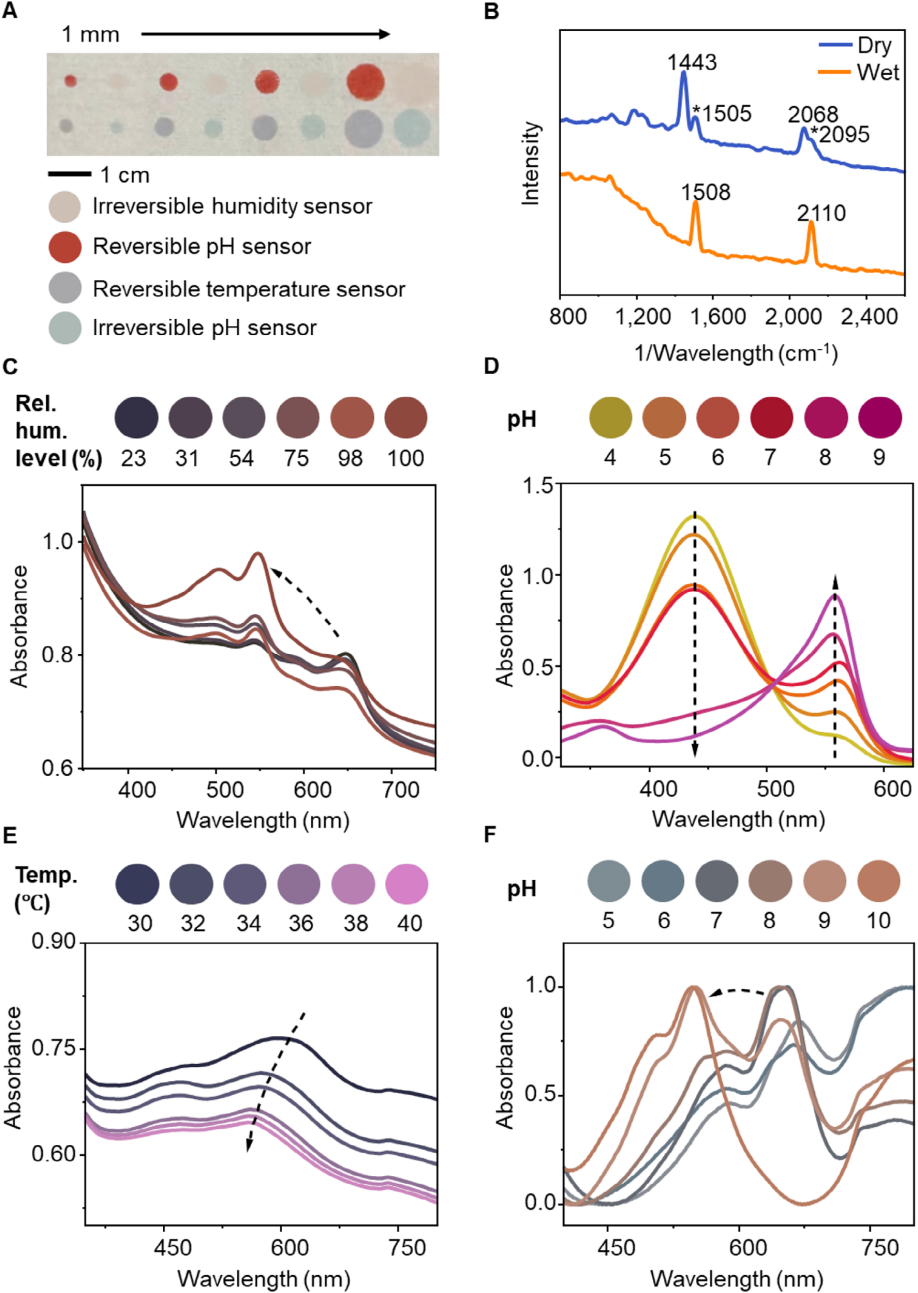
Characterization of the colorimetric sensors. A) Layout and composition of the colorimetric sensing array printed on a nonwoven dressing. The humidity sensor shows color before UV activation. B) Raman spectra of the humidity sensor in dry (blue) and wet (red) conditions. Peaks marked with * correspond to the partially converted red phase. C–F) corresponding quantitative analysis conducted by UV–vis spectroscopy across the sensing range of C) humidity sensor, D) reversible pH sensor, E) temperature sensor, and (F) irreversible pH sensor.

**Figure** **2**A shows the printed colorimetric sensing array fabricated on a nonwoven substrate, featuring sensors with diameters ranging from 1 to 3 mm. This array consists of four model sensor types—reversible and irreversible pH sensors, a reversible temperature sensor, and an irreversible humidity sensor—designed to monitor critical wound environment parameters. The colorimetric sensing inks were adapted from previously reported materials and optimized to enhance rheological properties for printing, while maintaining their established biocompatibility.^[^ ^49^, ^50^, ^51^, ^52^, ^53^, ^54^ ^]^ Figure 2C–F highlights the optical responses of the sensors under different analyte concentrations, with visible color changes depicted across the spectrum. The irreversible humidity sensor is created by embedding a hydrochronic diacetylene monomer within a PDMS matrix (Figure S9, Supporting Information). After UV activation following printing, the sensor undergoes a color change from light pink to blue due to polymerization (Figure S10, Supporting Information). The humidity‐induced color response is triggered by the hygroscopic groups in the polydiacetylenes (PDA), which cause electronic shifts in the conjugated polymer backbone as humidity levels change (Figure S11A, Supporting Information). Movie S3 (Supporting Information) demonstrates the different reaction speeds when water is applied to a pure PDA‐coated glass slide and a PDMS/PDA‐coated glass slide. Encapsulation within the PDMS matrix stabilizes the PDA, ensuring consistent and controlled color transitions (Figure S11B, Supporting Information). The humidity sensor demonstrated a response time of ≈15 min following moisture exposure. Figure 2B presents Raman spectroscopy data confirming the structural transitions of PDA, as it shifts from the blue phase to the red phase upon exposure to water. Figure 2C illustrates the sensor's color change from blue to red as humidity increases. The absorbance spectra reveal a progressive red shift with an observable peak shift from 23% to 100% relative humidity level, demonstrating the sensor's distinct color transition to varying moisture levels.

The reversible pH sensor is synthesized using a chitosan‐based polymeric dye, where phenol red (PR) is covalently grafted onto the chitosan backbone (Figure S12, Supporting Information). This covalent attachment enhances color stability and reduces dye leaching, while the chitosan matrix ensures biocompatibility.^[^ ^50^, ^55^ ^]^ The sensor displays pH‐dependent color changes through the protonation and deprotonation of PR groups (Figure S13, Supporting Information). In acidic conditions, the sensor appears yellow due to the protonated state, transitioning to red in alkaline conditions as deprotonation occurs. Figure 2D illustrates the sensor's dynamic range, covering pH 4 to 9, which corresponds to the range typically observed in wound exudates. Movie S4 (Supporting Information) showcases the reversible pH sensing capability, demonstrating a printed butterfly design on nonwoven fabric as it transitions repeatedly between pH 4 and pH 9 buffer solutions. The color change occurs rapidly, with a response time of less than one minute upon pH shifts, enabling timely detection of wound condition changes.

The reversible temperature sensor is developed by blending thermochromic powders into a PDMS ink formulation (Figure S14A, Supporting Information). By incorporating three thermochromic powders with different activation thresholds, the sensor is tuned to a temperature‐sensitive range of 30 to 40 °C, which is relevant for wound environments (Figure S14B, Supporting Information). Figure 2E shows the temperature‐dependent color transitions, demonstrating accurate responsiveness within the physiological range. Movie S5 (Supporting Information) highlights the rapid and reversible response capability of the temperature sensor through multiple cycles of color change, with visible transitions occurring within 1minute of temperature fluctuation.

The irreversible pH sensor is constructed using chitosan paste embedded with PDA vesicles (Figure S15, Supporting Information). Protonation and deprotonation alter the electronic structure and conformation of the PDA backbone, disrupting π‐electron conjugation and inducing a color shift in the vesicles (Figure S16, Supporting Information). At low pH, the sensor retains a stable blue color, indicating a protonated and intact conjugated structure. As the pH rises, deprotonation causes electrostatic repulsion, disrupting conjugation and transitioning the color to red, eventually turning orange at higher pH levels. Figure 2F shows this irreversible color change from blue (pH 6) to brown (pH 8) and orange (pH 10). The full color transition occurs within ≈15 min after pH exposure, supporting its practical usability in wound environments. Movie S6 (Supporting Information) demonstrates the irreversible pH sensing mechanism with the same design.

To confirm the precision of these colorimetric responses, Figure S17 (Supporting Information) presents CIE 1931 color space data, highlighting the chromatic accuracy of these transitions. To further validate sensor reproducibility and environmental resilience, we assessed the consistency and stability of the printed arrays under both production‐scale and stress‐test conditions. Figure S18 (Supporting Information) shows the RGB signal uniformity across more than 5 meters of R2R fabricated smart dressing rolls on a nonwoven substrate, comprising 20 imprints, each containing 176 sensing units. Six units per imprint were randomly selected for RGB analysis, revealing minimal variation across pH, temperature, and humidity sensors—demonstrating excellent reproducibility and uniformity throughout the roll. To assess robustness under environmental fluctuations, sensors were subjected to repeated thermal cycling (−20 to 50 °C) and alternating dry–wet humidity conditions. As shown in Figure S19 (Supporting Information), the reversible pH and temperature sensors maintained consistent and repeatable color transitions across multiple cycles, while the irreversible pH and humidity sensors retained stable post‐response RGB values, as designed.

To assess the functional integrity of the smart dressing under practical conditions, we evaluated its breathability and mechanical durability. As shown in Figure S20 (Supporting Information), the pH sensors exhibited a visible color change in response to ammonia vapor within 15 s, confirming effective gas permeability. Mechanical testing (Figure S21, Supporting Information) demonstrated that the printed dressings retained flexibility and stretchability comparable to uncoated substrates, with no visible surface defects on the sensor region up to 10% strain. Since the sensing elements occupy only a portion of the total surface area, the dressing's overall compliance is largely preserved. The modularity of the R2R process also allows the sensor layout to be adjusted to meet specific mechanical or clinical requirements. These results confirm that the sensors preserve structural integrity and functional reliability across variable environmental conditions, reinforcing the scalability and durability of our R2R‐fabricated platform for real‐world wound monitoring applications.

### 2.3 Characterization of Antifouling Effect

**Figure 3:**
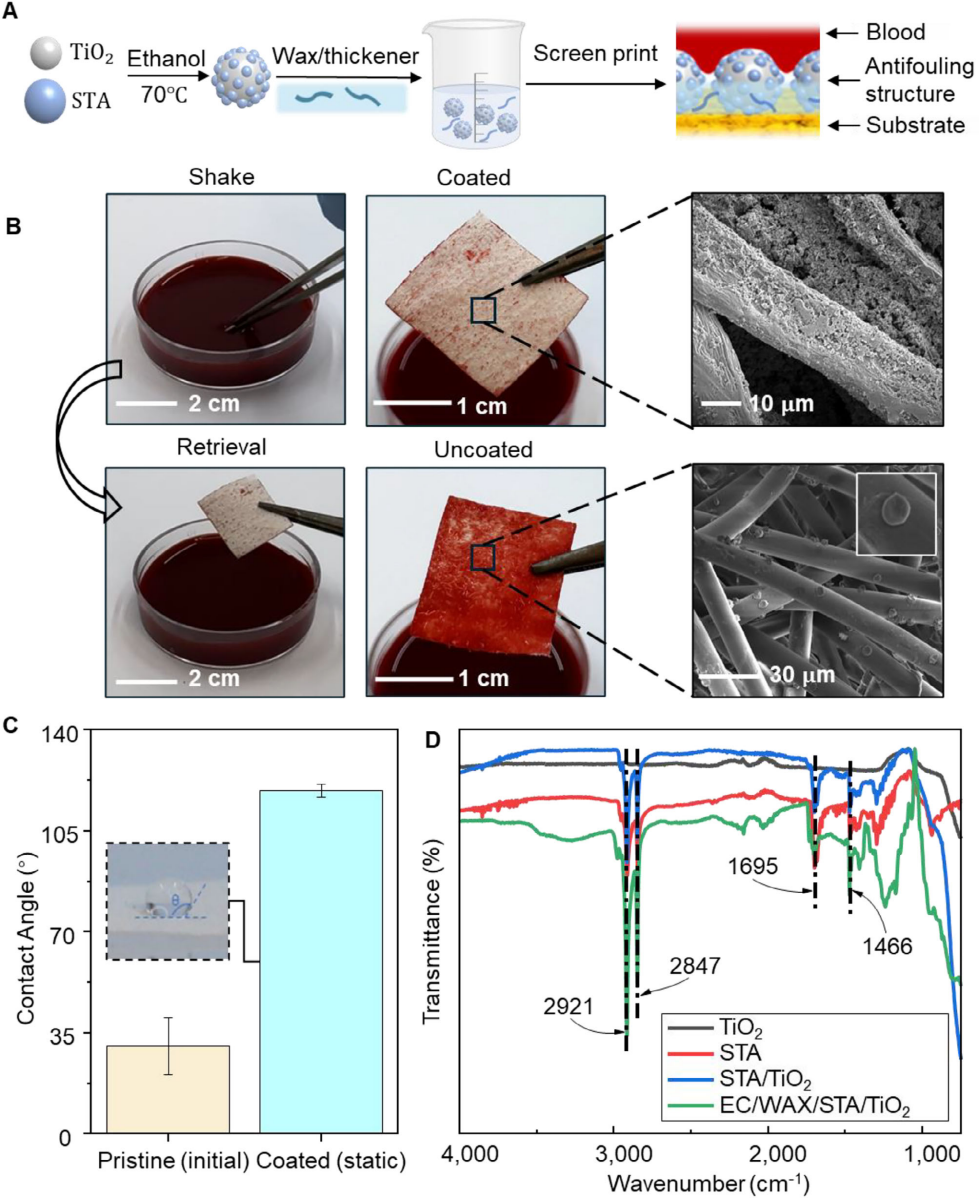
Characterization of the antifouling coating. A) Schematic illustration of the antifouling coating preparation process. B) Blood repellency test comparing coated and uncoated nonwoven dressing samples after immersion in whole blood, with corresponding SEM images highlighting microstructural differences. C) Water contact angle comparison of pristine and coated nonwoven dressing surfaces, pristine dressing is evaluated with the initial water contact angle and coated dressing is evaluated with the static contact angle. D) FTIR spectra confirming hydrophobic surface modification of STA/TiO₂ nanoparticles in the antifouling coating.

**Figure** **3**A depicts the preparation process for the antifouling coating, alongside a schematic illustrating the blood‐repellent behavior of the coated dressing fabric. STA/TiO₂ nanoparticles are synthesized by modifying titanium dioxide (TiO₂) nanoparticles with stearic acid (STA), forming rough microstructures crucial for achieving hydrophobicity. A screen‐printable ink is prepared by combining STA/TiO₂ nanoparticles with carnauba wax and ethyl cellulose (EC) in an ethanol solution. The robust blood‐repellent performance of the coated dressing fabric is due to the hybrid micro/nano‐structured hydrophobic surface, which maintains a protective air layer when exposed to blood, which minimizes blood interference in wound exudate and ensures accurate color readings. To evaluate the blood‐repellent properties, we immersed a nonwoven dressing sample—coated with the antifouling layer on its top surface, leaving the bottom uncoated—in a vigorously agitated porcine blood bath (Figure 3B). The uncoated bottom surface was quickly wetted by the blood, whereas the antifouling‐coated top surface effectively resisted contamination, remaining largely unaffected. SEM images highlight the difference in surface morphologies, showing significantly reduced erythrocyte adhesion on the antifouling‐coated surface due to the STA/TiO₂ microstructure. The static contact angle measured on the coated nonwoven dressing surface was *Θ* = 119° ± 2.3°, confirming its hydrophobic nature (Figure 3C).

Figure 3D shows the infrared spectra of unmodified TiO₂ nanoparticles, STA/TiO₂ nanoparticles, and the EC/WAX/STA/TiO₂ coating. For STA/TiO₂ nanoparticles, the absorption peaks at 2921 and 2847 cm⁻¹ correspond to the stretching vibrations of ─CH₃ and the symmetric stretching of ─CH₂ within C─H bonds, respectively. These peaks confirm the successful grafting of low‐surface‐energy groups (─CH₃ and ─CH₂) onto the TiO₂ surface, imparting hydrophobicity.^[^ ^56^ ^]^ The EC/WAX/STA/TiO₂ coating retains these characteristic peaks, indicating that the addition of EC and wax does not compromise the STA‐induced hydrophobic properties. The screen‐printable antifouling ink demonstrates broad applicability across various dressing materials. Figure S22 (Supporting Information) displays the uniform antifouling morphology on cotton, gauze, and nonwoven dressings, where the STA/TiO₂ microstructure effectively coats the textile surface, enhancing surface roughness. For further evaluation, 20 µL blood drops were applied to the coated dressing materials for sliding tests (Figure S23A, Supporting Information) and observed over one hour as they evaporated (Figure S23B, Supporting Information). On surfaces tilted at 10° and 25°, the blood drops easily slide off the nonwoven and gauze dressings, while some residue remained on the cotton dressing due to its inherent surface roughness. After one hour, minimal blood stains were left, which were easily wiped away.

To evaluate breathability, bromothymol blue (BTB)‐dyed water droplets were applied, transitioning from yellow to blue upon exposure to ammonia, indicating excellent air permeability (Figure S24, Supporting Information). The mechanical durability of the coated fabric was assessed through an abrasion test (Figure S25A, Supporting Information). While a slight decrease in the water contact angle was observed with increasing abrasion cycles, the angle remained above 115° even after 20 cycles, demonstrating sustained hydrophobicity (Figure S25B, Supporting Information). SEM images taken after 10 and 20 abrasion cycles confirmed that the surface roughness was preserved, validating the mechanical stability of the antifouling coating (Figure S25C, Supporting Information). These findings underscore the coated fabric's suitability for wound dressing applications, offering excellent air permeability to promote breathability critical for wound healing, alongside sustained hydrophobicity and mechanical durability for reliable, long‐term antifouling performance. Figure S26 (Supporting Information) presents a cross‐sectional view of the smart wound dressing, illustrating the sensor layers printed on the outer surface of the dressing. This configuration preserves the inner wound‐contacting side for standard wound care procedures while maintaining porosity beneath the sensor layer. The uncoated fabric substrate enables wound exudate to diffuse upward and activate the sensors, whereas the antifouling layer, applied only above the cured sensor, prevents surface fouling without impeding biomarker detection. To test the efficacy of the antifouling layer and ensure sensor visibility in the presence of blood‐contaminated wound exudate, an artificial wound fluid (AWF) with pH 9 and a high blood concentration was used (Figure S27A, Supporting Information). The smart wound dressing patch was submerged in AWF for 30 min and then placed on a 40 °C hot plate for color calibration. As depicted in Figure S27B (Supporting Information), the color calibration results accurately align with the AWF status, exhibiting minimal errors of less than 5%, confirming the antifouling layer's effectiveness and the clear visibility of the sensors even in blood‐contaminated conditions.

### 2.4 Cell Viability and In Vivo Evaluations in Mice Models

**Figure 4:**
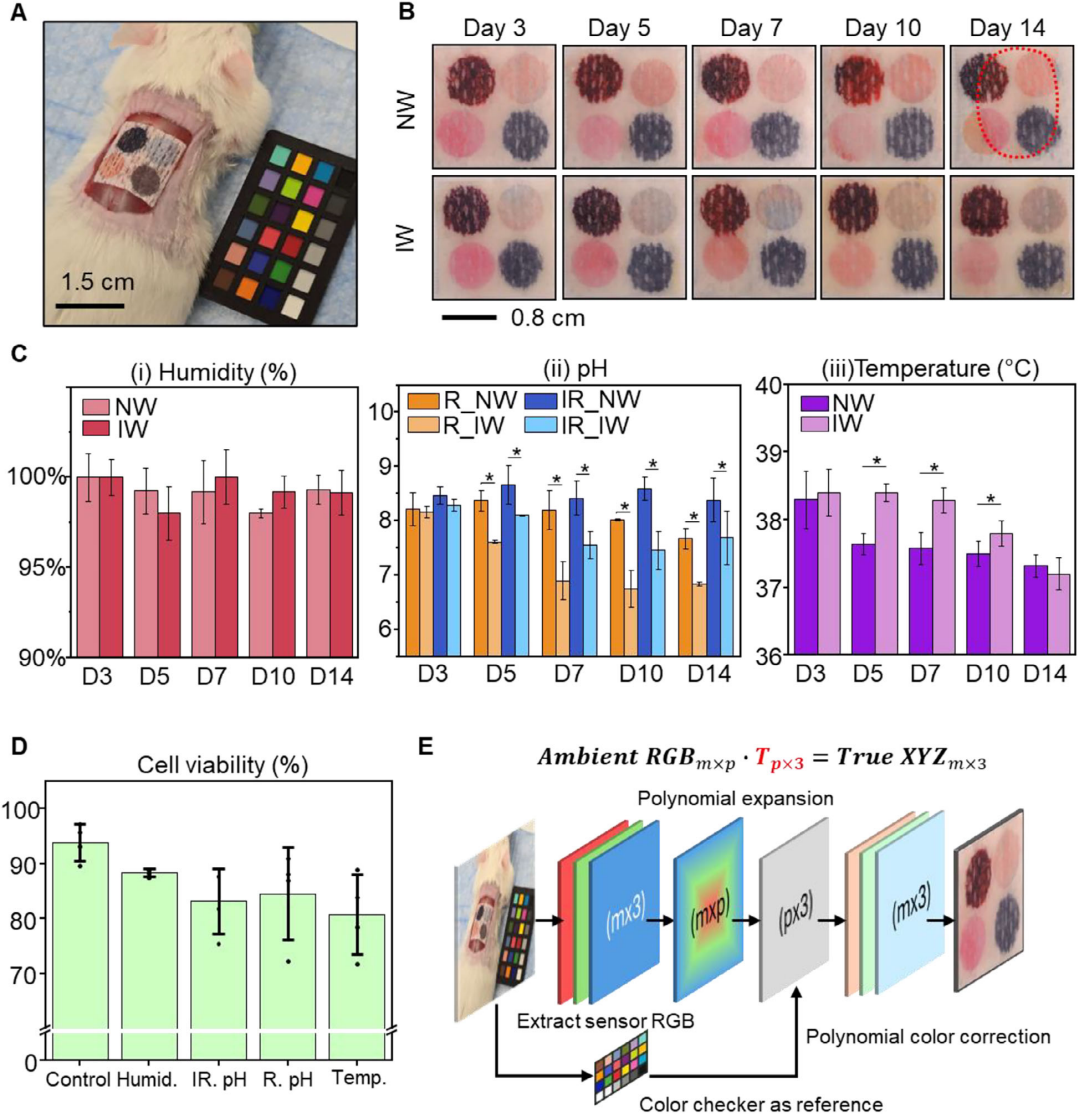
In vivo evaluation of smart wound dressing in mouse models. A) Experimental setup showing the smart dressing, fabricated on a gauze substrate, applied to a mouse wound with a color checker included for image calibration. B) Images of the detached sensor patches on non‐infected and infected wounds over 14 days, with visual color changes indicating wound status. (Top left – humidity sensor, top right – irreversible pH sensor, bottom left – reversible pH sensor, bottom right – temperature sensor. The temperature sensor shows no color change due to detachment). C) Quantitative analysis of i) humidity, ii) pH, and iii) temperature in non‐infected (NW) and infected (IW) wounds over time. Data are presented as mean ± SD. For each group, four mice were used, and images captured under both 4000 and 5500 K lighting conditions were analyzed. Sample sizes: NW group, *n* = 8 at all‐time points; IW group, *n *= 8 (Days 3 and 5), *n* = 6 (Day 7), and *n *= 4 (Days 10 and 14), due to the sacrifice of two mice at later stages from severe infection. Asterisks (*) denote statistically significant differences between NW and IW groups (*p < 0.05; Welch's *t*‐test). (D) Cell viability assay of each sensor. E) Polynomial color correction model for accurate sensor color interpretation using ambient RGB and color checker reference.

**Figure** **4**A shows the setup for the in vivo study, in which a smart wound dressing patch fabricated on a gauze substrate is applied to a dorsal wound on the mouse, with a color checker included for image analysis and color calibration. This substrate was chosen due to the small wound size and limited exudate production in the mouse model, as gauze requires minimal moisture to activate visible sensor color changes. To evaluate the effectiveness of the smart wound dressing for point‐of‐care monitoring, a 14‐day study was conducted using both infected and non‐infected mouse wound models. The experiment involved eight mice, labeled S01 to S08, with S01 to S04 in the non‐infected (NW) group and S05 to S08 in the infected (IW) group. Each mouse had a full‐thickness dorsal wound, with the infected group inoculated with *Pseudomonas aeruginosa* (*P. aeruginosa*). The smart dressings were secured over the wounds using Tegaderm to enhance exudate collection. Smart dressings were replaced daily throughout the 14‐day study to ensure consistent sensor performance and to capture dynamic changes in the wound microenvironment. This routine enabled each irreversible sensor to provide a distinct, time‐integrated snapshot of wound conditions over each 24‐hour period. Photographs of the sensor patches were taken on Days 3, 5, 7, 10, and 14, using the color checker for accurate quantification of biomarker changes.

Figure 4B illustrates the color progression of the sensors, capturing their response to wound status. For imaging purposes, the sensor patches were detached to provide a clear view of the sensing regions. As a result, temperature‐induced color changes are not visible due to the absence of thermal activation during image capture. Despite this, distinct color transitions in both the irreversible and reversible pH sensors are observed, reflecting dynamic shifts in the wound microenvironment. Figures S28 and S29 (Supporting Information) provide detailed color records for each mouse on the respective days. Figure S30 (Supporting Information) highlights the differences in wound healing between infected and non‐infected wounds. Non‐infected wounds maintained a stable pink color, with visible wound contraction beginning on Day 7, indicating normal healing. In contrast, infected wounds exhibited a transition from pink to intense yellow and pale hues, suggesting ischemia, accompanied by swollen and enlarging wound edges indicative of severe infection and delayed healing. Figure 4E illustrates the color regression model used for color correction, ensuring precise and refined analysis. Figure 4C presents the humidity, pH, and temperature fluctuations after color calibration with the regression model over the 14‐day experiment. The irreversible humidity sensor functions as a cumulative biomarker, tracking overall moisture exposure to confirm sensor activation and assist in exudate management rather than capturing real‐time fluctuations. As shown in Figure 4C‐i, all sensor patches demonstrated sufficient wettability, with minimal differences observed within the measurement window, likely due to the use of Tegaderm to enhance moisture retention. Notably, the sensors on infected wounds reached saturation more quickly than those on noninfected wounds, indicating greater moisture accumulation in infected wounds.

Both wound types exhibited an alkaline pH on Day 3, consistent with the onset of early inflammation. In noninfected wounds, pH showed a slight increase from Day 3 to Day 5, marking the transition into the proliferation phase, followed by a gradual decline beginning on Day 7. The pH then stabilized around pH 7 during Days 10 to 14, corresponding to the remodeling phase. In contrast, infected wounds demonstrated a continuous decrease in pH from Day 3 onward, as *P. aeruginosa* colonized the wound bed, formed biofilms, and established acidic microenvironments—particularly during the acute infection phase (Days 5–7). pH levels stabilized between Days 10 and 14, indicating the onset of the resolution phase. The irreversible pH sensor followed the same temporal trend for both wound types, though it recorded slightly higher absolute values, reflecting peak pH during sensor wear. Welch's *t‐*test revealed statistically significant differences in pH between infected and noninfected groups beginning on Day 5 for both sensor types (*p* < 0.05) (Figure 4C‐ii). Temperature profiles also differed: both groups peaked in surface temperature on Day 3. Noninfected wounds showed a continuous decline thereafter, reaching ≈37.1 °C by Day 14, consistent with the resolution of inflammation. Infected wounds, however, exhibited sustained elevated temperatures through Day 7, followed by a decline beginning on Day 10—coinciding with the attenuation of acute infection and potential onset of ischemia (Figure 4C‐iii).

The biocompatibility of the smart wound dressing was evaluated to identify any potential adverse cellular responses. Sensor patches measuring 1.5 cm × 1.5 cm were screen‐printed with all sensing materials, sterilized under UV light, and seeded with NIH/3T3 cells. The cells were cultured on the patches at 37 °C with 5% CO₂ until confluency. Details of the cell viability assay are provided in the Experimental Section. Figure 4D presents the results, showing cell viability remained above 80% throughout the assay. Fluorescence images of live and dead cells are displayed in Figure S31 (Supporting Information), further confirming the biocompatibility of the smart wound dressing.

## 3 Discussion

By pioneering the first application of R2R technology for embedding colorimetric sensors in wound care, this study presents a breakthrough in the large‐scale fabrication of smart wound dressings. This scalable and cost‐effective approach enables real‐time monitoring of critical wound biomarkers, including pH, temperature, and humidity, without the need for complex instrumentation. By demonstrating the feasibility of high‐throughput manufacturing, this work establishes a new standard for mass‐producing advanced wound monitoring systems, bridging the gap between laboratory research and clinical application. The smart dressing offers an accessible and user‐friendly solution for both patients and caregivers, providing immediate visual feedback on wound status. Its adaptability to various dressing materials and wound types underscores its versatility, ensuring broad applicability in personalized wound management. By detecting critical indicators such as infection, inflammation, or ischemia, the device facilitates timely interventions and supports personalized wound care. Its cost‐effective, point‐of‐care design reduces the frequency of clinical visits, alleviating pressure on healthcare systems while enabling effective home‐based treatment. Additionally, the platform shows potential for specialized applications, including diabetic ulcers, burns, and postoperative wound care.

This work also sets the stage for extending the capabilities of R2R‐fabricated devices by incorporating additional biomarkers tailored to specific wound conditions or chronic diseases. For instance, integrating glucose sensors could address the unique requirements of diabetic wound management,^[^ ^57^ ^]^ while lactate sensors could provide insights into tissue oxygenation and metabolic activity, particularly in ischemic wounds.^[^ ^58^ ^]^ Inflammatory biomarkers such as C‐reactive protein (CRP) or interleukin‐6 (IL‐6) could enhance the device's ability to monitor wound progression and systemic inflammation.^[^ ^59^ ^]^ Oxygen saturation sensors could improve assessments of vascular health,^[^ ^60^ ^]^ and electrolyte sensors could aid in managing fluid balance in wounds with high exudate output, such as burns.^[^ ^61^ ^]^ These advancements would significantly expand the diagnostic utility of the platform, enabling precision wound care.

While this study represents a significant advancement, several challenges remain to be addressed. The R2R fabrication process, though capable of high‐throughput production, requires further refinement to maintain consistent printing quality during extended manufacturing runs. Ensuring the stability of colorimetric inks under prolonged shear forces and airflow is a key priority. Additionally, improving their sensitivity to detect low‐concentration biomarkers and enabling multiplexed monitoring of multiple indicators will enhance clinical applicability. This may involve developing novel bioinks with greater functionality and robustness. Future work should also prioritize biocompatibility and sustainability by exploring biodegradable substrates and inks, while minimizing the environmental impact of disposable devices. As part of this effort, comprehensive long‐term biocompatibility assessments—including systemic toxicity, immune response, and degradation analysis—are planned as a critical next step toward clinical translation. In addition, extended in vivo studies, including those involving human tissue models, will be necessary to ensure long‐term safety.

In conclusion, this study represents a pioneering application of R2R fabrication in wound monitoring, providing a scalable, cost‐effective, and versatile platform for advanced wound care. By addressing current challenges, incorporating additional biomarkers, and prioritizing sustainability, this technology has the potential to revolutionize wound management practices, improve patient outcomes, and set a new standard for environmentally conscious medical devices.

## 4 Experimental Section

### Materials

Chitosan, phenol red (PR), and glacial acetic acid were obtained from Sigma Aldrich. Sodium hydroxide, formaldehyde (37% w/w), dimethylformamide, 10,12‐docosadiynedioic acid (DCDA), 10,12‐pentacosadiynoic acid (PCDA), hydroxyethyl cellulose, ethyl cellulose, nano titanium dioxide (rutile TiO₂, 100 nm particle size), stearic acid, carnauba wax, and ethanol were sourced from Fisher Scientific. The SYLGARD 184 silicone elastomer kit was obtained from Ellsworth Adhesives. Thermochromic powders were acquired from Atlanta Chemical Engineering, and nonwoven fabric rolls, cotton, and gauze dressings were purchased from Amazon.

### R2R Screen Printing

Screen masks were procured from NBC Meshtec, and nonwoven fabric dressing served as the substrate, attached to a PET roll. Dummy prints ensured alignment and quality before initiating automated printing. The reversible pH sensor layer was printed first, dried in a convection dryer at 80 °C for 10 min, and rewound for subsequent layers. Irreversible pH, humidity, and temperature sensor layers were printed and dried sequentially at 80 °C for 10, 30, and 25 min, respectively. The antifouling coating was printed last and dried for 5 min at 80 °C. The humidity sensor was UV activated after printing at 254 nm for 60 s. Each layer's printing and curing process took ≈20 min for 100 imprints over 60 meters of dressing roll.

### Ink Formulation

Chitosan stock solution was prepared by dissolving 1% w/v chitosan in 1% acetic acid, followed by continuous stirring at room temperature overnight to ensure complete dissolution. The PDMS stock paste was prepared by mixing SYLGARD 184 elastomer base and curing agent at a 10:1 mass ratio.

The reversible pH sensing ink was formulated using a CS‐PR polymeric dye synthesized via a Mannich reaction, as previously reported by Chalitangkoon et al.^[^ ^50^ ^]^ Specifically, 934 mg of phenol red was first dissolved in 50 mL of DMF and mixed with 232 mg of formaldehyde, then added to 100 mL of the chitosan stock solution. The mixture was stirred at 60 °C for 24 h. Following this, 33.4 mL of 0.5 m NaOH was added dropwise to precipitate the polymeric dye. The precipitate was filtered, washed three times with ethanol, redispersed in deionized water, and centrifuged at 6000 rpm to remove unreacted dye. After drying at 60 °C, the product was ground into a fine powder. For ink preparation, 1% w/v of the polymeric dye was dissolved in 1% acetic acid and mixed 1:1 by volume with the chitosan stock solution. To enhance printability, 1 wt.% hydroxyethyl cellulose was added.

For the irreversible pH sensing ink, 2 mm of dicyandiamide (DCDA) was dissolved in a 1:1 ethanol–water solution and sonicated for 1 h. The solution was poured into a Petri dish and polymerized under 254 nm UV light for 60 s. The resulting polymer was mixed with the chitosan stock solution in a 1:2.5 volume ratio and stirred at room temperature. To improve the ink's rheological behavior, 1 wt.% hydroxyethyl cellulose was incorporated.

The irreversible humidity sensing ink was based on hydrochromic PCDA, synthesized following a method reported by Lee et al.^[^ ^53^ ^]^ Briefly, 750 mg of cesium hydroxide (5 mmol) was dissolved in 0.4 mL of water and added to 9.6 mL of THF containing 1.87 g of PCDA (5 mmol). The mixture was stirred at room temperature for 1 h. The final ink was prepared by incorporating the synthesized hydrochromic PCDA into the PDMS stock paste at 5 wt.%, using a Thinky mixer for uniform dispersion.

For the reversible temperature sensing ink, thermochromic dyes with activation thresholds of 31, 35, and 38 °C were blended at a ratio of 3.5:1:0.5 and added to the PDMS stock paste at 1 wt.% total. The mixture was homogenized using a Thinky mixer to ensure consistent dye dispersion.

The antifouling coating ink was prepared by mixing TiO₂ and stearic acid at a 6:1 mass ratio in ethanol, followed by ultrasonication at 70 °C for 1 h. The mixture was centrifuged and dried at 80 °C. The resulting STA/TiO₂ sediment was ground and stored for later use. To formulate the coating ink, 2 g of STA/TiO₂ and 0.2 g of carnauba wax were dispersed in 40 mL of ethanol and magnetically stirred at room temperature for 30 min. To improve print quality, 1 wt.% ethyl cellulose was added to the mixture.

### Characterizations of the Sensing Material

The Cary 6000i UV–vis–NIR spectrophotometer was used to monitor color transitions of sensing films under varying environmental conditions. Sensing films for UV–vis experiments were prepared by drop casting the sensing inks. pH sensing films were immersed in buffer solutions for 10 min before testing. Humidity‐sensitive films were conditioned in sealed, custom glass chambers containing pure water, silica gel, or saturated salt solutions (Table S3, Supporting Information) to achieve controlled relative humidity levels, with all samples equilibrated for at least 1 h at 23 °C prior to photographic or reflectance measurements to ensure consistency and accuracy. Temperature tests ranged from 30 to 40 °C. Raman spectroscopy, using a WITec Alpha 3000 Microscope with 633 nm laser excitation, analyzed the structural changes in humidity sensing films before and after water spray. Rheological behavior of the inks was measured using a TA Instruments DHR3 Rheometer.

### Characterizations of the Antifouling Coating

SEM imaging was performed with a Hitachi S‐4800 Field Emission SEM on sputter‐coated dressing samples, and FTIR analysis using a Nicolet 6700 confirmed chemical states of the antifouling material. Wettability tests using a Tantec Contact Angle Micrometer evaluated pristine and coated dressing surfaces. Mechanical stability was assessed with abrasion resistance tests using 1000‐mesh sandpaper under a 200 g load, recording contact angles after 10 cm strokes at 5 cm s^−1^. Blood‐repellency tests included submerging coated and uncoated dressing sides in porcine blood for comparison, applying 20 µL droplets for 1 h before wiping, and conducting sliding tests. Air permeability was verified using BHB‐dyed water droplets atop coated dressing samples covering sealed vials of ammonia and water. Artificial wound fluid (AWF) was prepared by diluting whole porcine blood with a buffer solution to achieve a final pH of 9. Dressing patches measuring 15 mm × 15 mm were soaked in the AWF for 30 min to stabilize the color. After soaking, the patches were placed on a 40 °C hot plate for color calibration. The absolute percentage error of the predicted biomarker levels was calculated by:

(1) $$Error\;\left(\%\right)=\left|\frac{P_{i}-A_{i}}{A_{i}}\right|$$

where *P_i_* is the predicted value of a certain biomarker *i*; and *A_i_* is the actual value.

### Cell Viability Assay

NIH/3T3 cells were cultured in Dulbecco's Modified Eagle Medium (DMEM), which contained 10% fetal bovine serum and 1% penicillin‐streptomycin. The sensor patches were cut to a size of 15 mm × 15 mm and put in the 6‐well culture plate. The samples were sterilized by UV light in the biological safety cabinet for 30 min. 0.3 × 10^6^ cells were seeded in each well and cultured in an incubator under 37 °C and 5% CO_2_ until the control group was a confluent monolayer. LIVE/DEAD cell viability assay kit (Invitrogen, USA) was used to analyze the viability of NIH/3T3 cells cultured on the sensor patches. A working solution containing calcein‐AM and ethidium homodimer‐1 was dropped on the cultured cells, and the cells were incubated for 30 min at room temperature. The images of live and dead cells were acquired by an LSM900 confocal microscope (Zeiss, Germany).

### Full‐Thickness Wound Model for Testing Colorimetric Sensor Patch

All procedures were conducted per the guidelines for the care and use of laboratory animals and were approved by the Institutional Animal Care and Use Committee (IACUC) of the University of Illinois at Urbana‐Champaign (Protocol No. 23 153). Healthy 8‐week‐old male CD‐1 mice (Charles River Laboratories, USA) were anesthetized using isoflurane and had their backs shaved. The size of a 12 mm × 12 mm full‐thickness wound was created on the back. For the infected wound condition, 0.1 mL of *P. aeruginosa* bacterial solution (10^6^ CFU mL^−1^) was inoculated on the wound. The sensor patch was applied directly to the wound, secured with Tegaderm (3 m, USA), and a bandage; the entire dressing setup was replaced daily. To investigate the feasibility of the colorimetric sensor patch, the patch was tested on both non‐infected and infected wounds for 14 days. The photograph of the attached sensor patches was taken on Days 3, 5, 7, 10, and 14 on the mice with color temperatures of 4000 and 5500 K, along with a color checker for color analysis. Additionally, images of detached sensor patches were also captured on the same days for comparison.

### Color Calibration of the Sensor Patch

A single sensor patch with a size of 1.5 cm × 1.5 cm was fabricated using screen printing. The sensor was tested under various environmental conditions, including pH buffers, a hot plate, and relative humidity vials, prepared as described above. Images of the colorimetric sensors, captured alongside a color checker (Macbeth ColorChecker), were taken using a smartphone (Samsung Galaxy S21) in PRO mode under white light with color temperatures of 4000 and 5500 K with auto white balance function ON. Regression‐based color correction, widely used with conventional color standards, is employed to enhance the accuracy of the sensor images. This method involved a mathematical relationship between the CIE XYZ tristimulus values under CIE illuminant E and the measured RGB values, expressed as:

(2) xT=y

where *x* is a 1 × 3 row vector containing the measured R, G, and B channel values, 𝑦 is a 1 × 3 row vector representing the corresponding CIE XYZ tristimulus values, and 𝑇 is a 3 × 3 transformation matrix that maps the RGB values to the CIE XYZ values under the given illuminant. Typically, the number of reference colors (e.g., *m* = 24) in standard color charts exceeds the number of unknowns (i.e., three color values). Incorporating *m* reference colors into Equation (2) transforms this underdetermined problem into an overdetermined system:

(3) $$X_{m\times 3}\;T_{3\times 3}=Y_{m\times 3}$$

where *T*_3 × 3_ can be computed using least‐squares regression. Fixed‐design linear regression, which included polynomial (or root‐polynomial) expansion terms of the R, G, and B values were employed. This approach enabled polynomial color correction. Specifically, five common types of polynomial or root‐polynomial expansions for RGB values were utilized. The acquired RGB vector *x*_1 × 3_ was expanded into $x_{1\times p}^{j}$, where $x_{1\times p}^{j}$ represents the 𝑗‐th type of expansion with 𝑝 total terms, as shown below:

(4) $$x_{1\times \left(p=4\right)}^{1}=\left[1,R,G,B\right]$$

(5) $$x_{1\times \left(p=10\right)}^{2}=\left[1,R,G,B,R^{2},G^{2},B^{2},RG,GB,BR\right]$$

(6) $$\begin{array}{ccc} x_{1\times \left(p=20\right)}^{3} & = & \left[1,R,G,B,R^{2},G^{2},B^{2},RG,GB,BR,R^{3},G^{3},B^{3},R^{2}G,\right.\\ & & \times \left.RG^{2},G^{2}B,GB^{2},B^{2}R,BR^{2},RGB\right] \end{array}$$

(7) $$x_{1\times \left(p=7\right)}^{4}=\left[1,R,G,B,\sqrt{RG},\sqrt{GB},\sqrt{BR}\right]$$

(8) $$\begin{array}{ccc} x_{1\times \left(p=14\right)}^{5} & = & \left[1,R,G,B,\sqrt{RG},\sqrt{GB},\sqrt{BR},\sqrt{R^{2}G},\sqrt{RG^{2}},\sqrt{G^{2}B},\right.\\ & & \times \left.\sqrt{GB^{2}},\sqrt{B^{2}R},\sqrt{BR^{2}},\sqrt{RGB}\right] \end{array}$$

using these expansions, Equation (3) is modified as follows:

(9) $$X_{m\times p}^{j}\;T_{p\times 3}=Y_{m\times 3}$$

this equation can be solved using the moore–penrose pseudoinverse:

(10) $$T_{p\times 3}={\left[X_{m\times p}^{j}\right]}^{+}\;Y_{m\times 3}$$

where (+) indicates the pseudoinverse operation. This transformation matrix maps RGB values acquired under specific conditions to the corresponding CIE XYZ values under illuminant E. Selecting an appropriate polynomial expansion depends on the device, lighting conditions, and file format, and must be tailored to each acquisition setup. Photographs of the attached sensor patches were taken on Days 3, 5, 7, 10, and 14, along with a color checker, under ambient light, and save in JPG format. The sensor patches remained in contact with the body for 60 min to ensure a full reaction. The acquired images were processed to extract RGB values from each sensor, which were then converted to CIE XYZ values through the color calibration. These calibrated values were compared against predefined standards to accurately determine the pH, temperature, and humidity levels measured by the sensors.

## Conflict of Interest

The authors declare no conflict of interest.

## Author Contributions

Z.W., Y.A., and S.K. contributed equally to this work. Y.L.K., H.K., and C.H.L. conceived the concept, planned the project, and supervised the research. Z.W., T.Y. and C.H.L. designed, fabricated, and characterized the smart wound dressing. Z.W., J.W., H.L., and C.H.L. characterized the antifouling property. Y.A., J.L., and H.K. designed and conducted the cell viability test and in vivo test on mice. Z.W., S.K., S.A.L., S.M.P., Y.L.K., and C.H.L. designed and conducted the color study for biomarker quantification. Z.W., Y.A., S.K., and C.H.L. wrote the manuscript. All authors commented on the paper.

## Supporting information



Supporting Information

Supplemental Movie 1

Supplemental Movie 2

Supplemental Movie 3

Supplemental Movie 4

Supplemental Movie 5

Supplemental Movie 6

## Data Availability

The data that support the findings of this study are available from the corresponding author upon reasonable request.
