# Supplementary material for: Roll‐To‐Roll Production of Smart Dressings for Wound Monitoring
Source: Adv Healthc Mater. 2025 Jul 22;14(27):e01998. doi: 10.1002/adhm.202501998 (PMC12538542; doi:10.1002/adhm.202501998)
Supplement: Supplementary file 1 — Supporting Information [file ADHM-14-0-s001.docx]

Supporting Information

**Roll-to-roll production of smart dressings for wound monitoring**

Ziheng Wang, Yujin Ahn, Semin Kwon, Tianhao Yu, Yumin Dai, Julia Walsh, Joo Hun Lee, Sang Mok Park, Seul Ah Lee, Murtuza Peerbhai, Chandan K. Sen, Hyowon Lee, Young L. Kim*, Hyunjoon Kong*, Chi Hwan Lee*

**The PDF file includes:**

Figs. S1 to S31

Tables S1 to S3

Legends for movies S1 to S6

**Other Supplementary Material for this manuscript includes the following:**

Movies S1 to S6


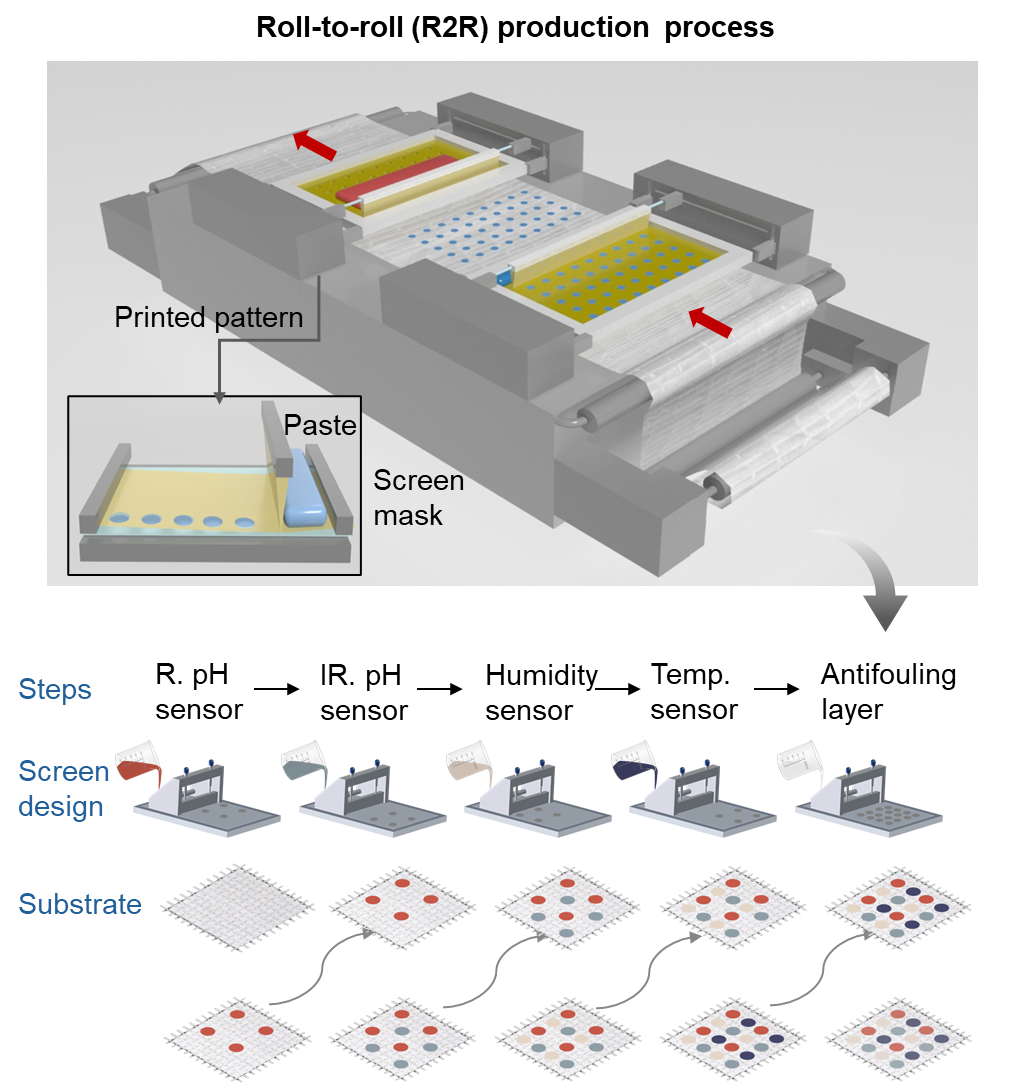


**Fig. S1.** Schematic of the step-by-step roll-to-roll screen printing process, illustrating the sequential ink deposition order.


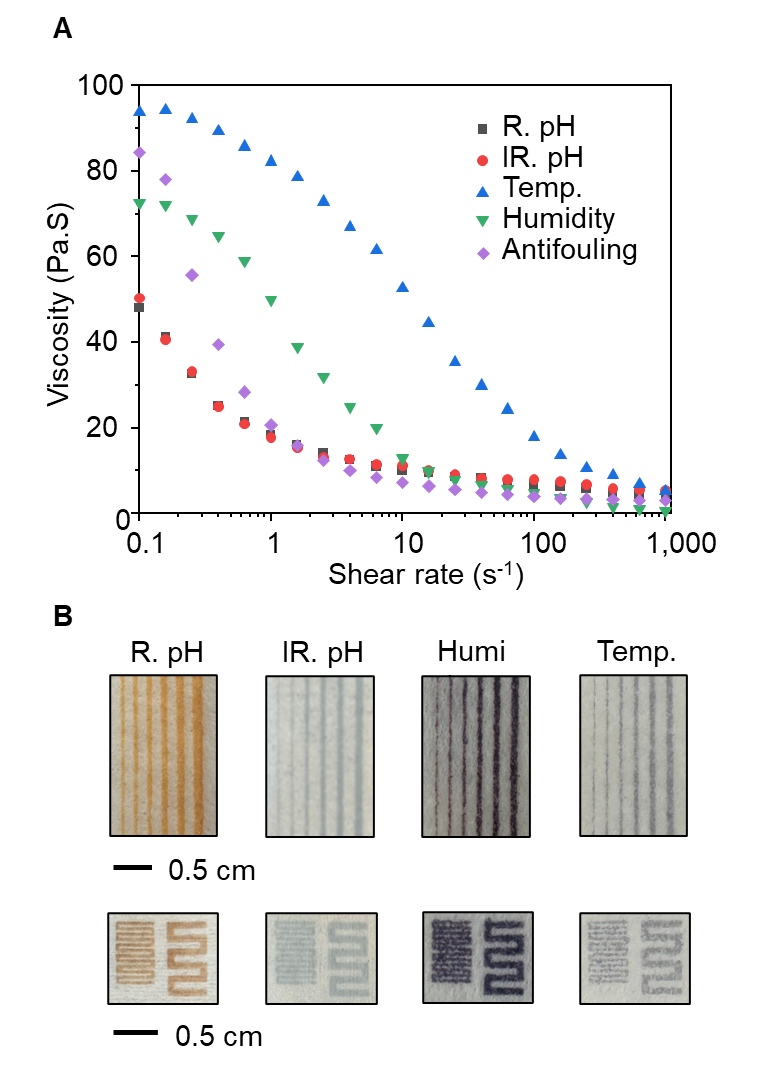


**Fig. S2.** Rheological behavior and print resolution of sensor inks. (A) Viscosity profiles of reversible pH, irreversible pH, temperature, humidity, and antifouling inks. (B) Print quality assessment for each sensor ink, with examples of printed straight and folded lines at varying widths printed on nonwoven substrates, demonstrating high-resolution patterning.


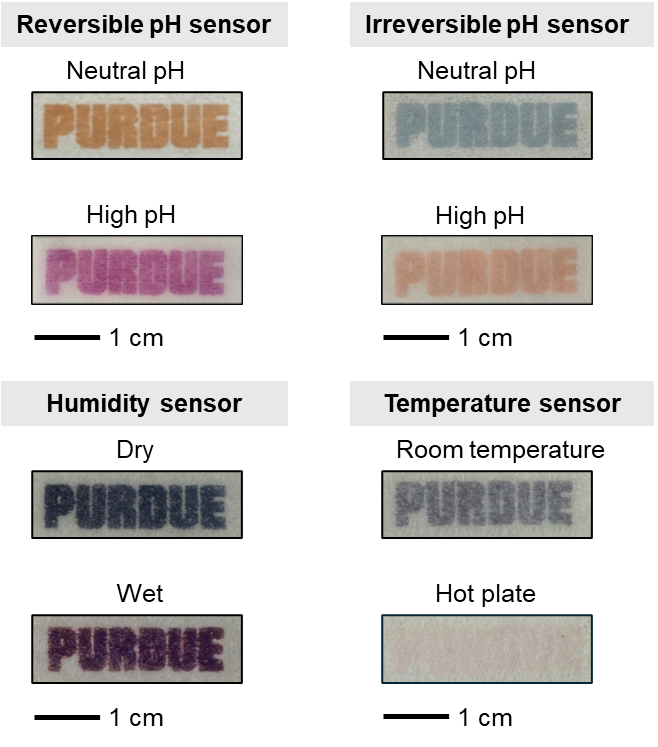


**Fig. S3.** Color change of screen-printed Purdue patterns using nonwoven substrate, demonstrating customizable and functional sensor design.

**
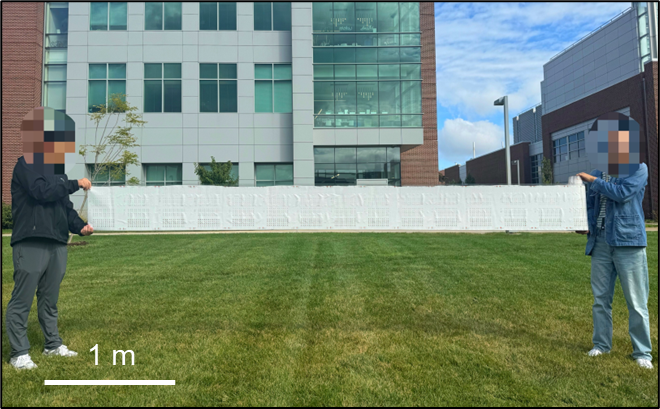
**

**Fig. S4.** Large-area image of the smart wound dressing roll fabricated on nonwoven substrate, highlighting the scalability and high-throughput capability of the roll-to-roll fabrication process.

**
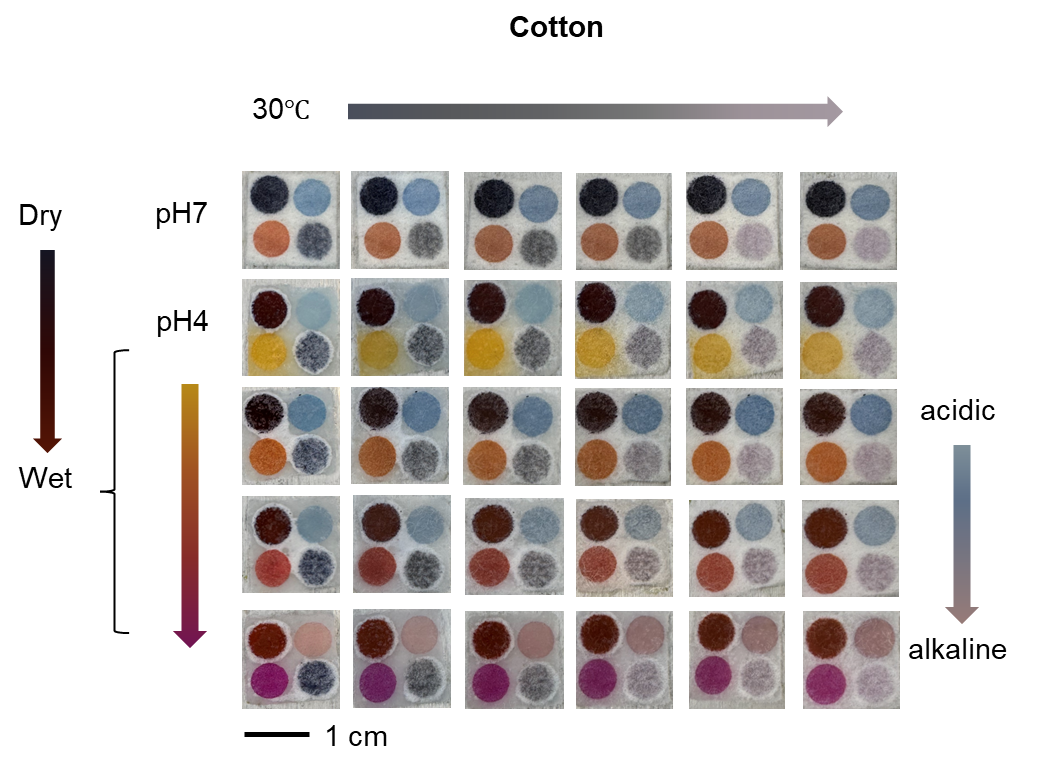
**

**Fig. S5.** Color map for smart cotton dressing. (Top left - humidity sensor, top right - irreversible pH sensor, bottom left - reversible pH sensor, bottom right - temperature sensor. Temperature sensor shows no color change due to detachment)

**
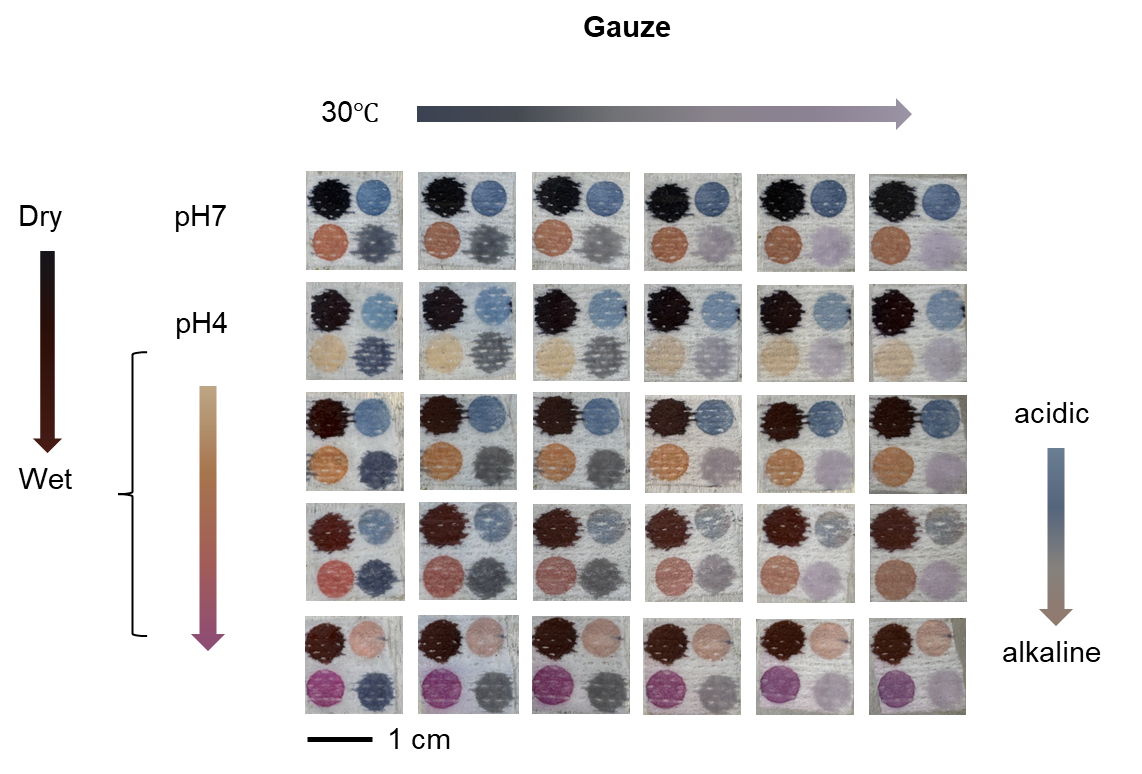
**

**Fig. S6.** Color map for smart gauze dressing. (Top left - humidity sensor, top right - irreversible pH sensor, bottom left - reversible pH sensor, bottom right - temperature sensor. Temperature sensor shows no color change due to detachment)

**
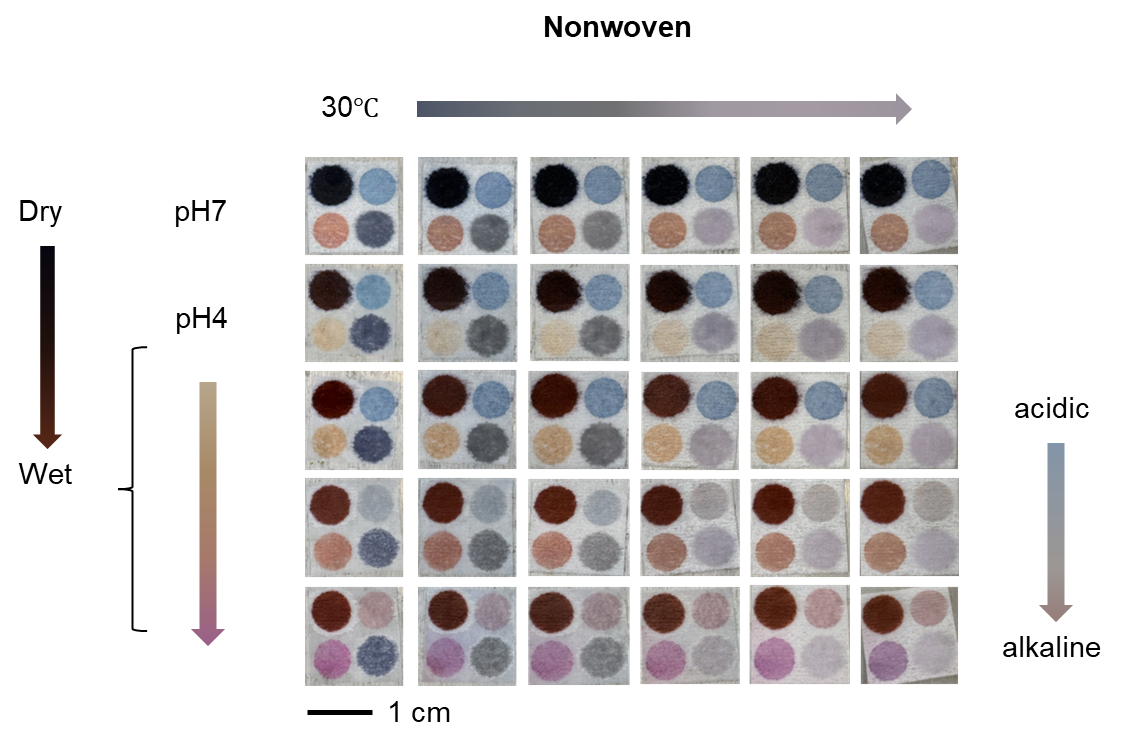
**

**Fig. S7.** Color map for smart nonwoven dressing. (Top left - humidity sensor, top right - irreversible pH sensor, bottom left - reversible pH sensor, bottom right - temperature sensor. Temperature sensor shows no color change due to detachment)

**
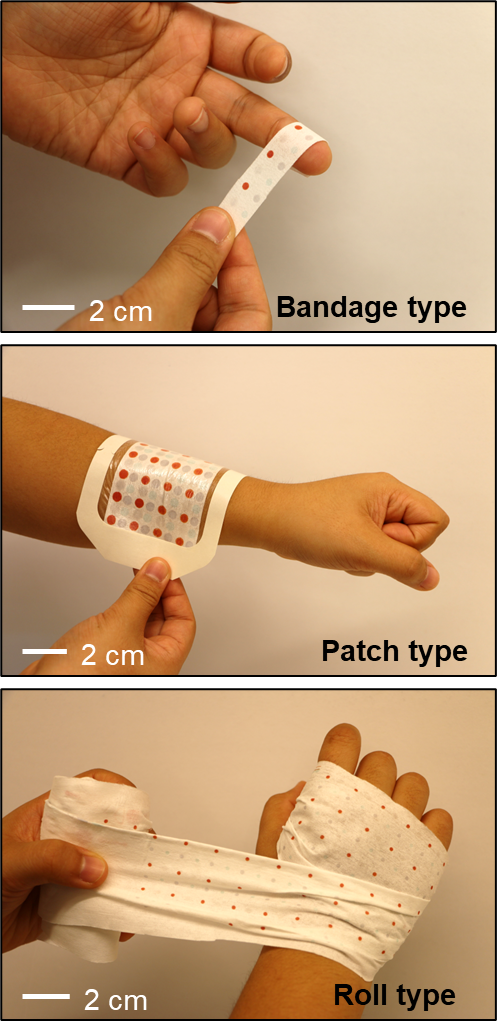
**

**Fig. S8.** Versatile form factors of the smart wound dressing fabricated on a nonwoven substrate, demonstrated in bandage, patch, and roll configurations to accommodate various application sites and coverage requirements.

**
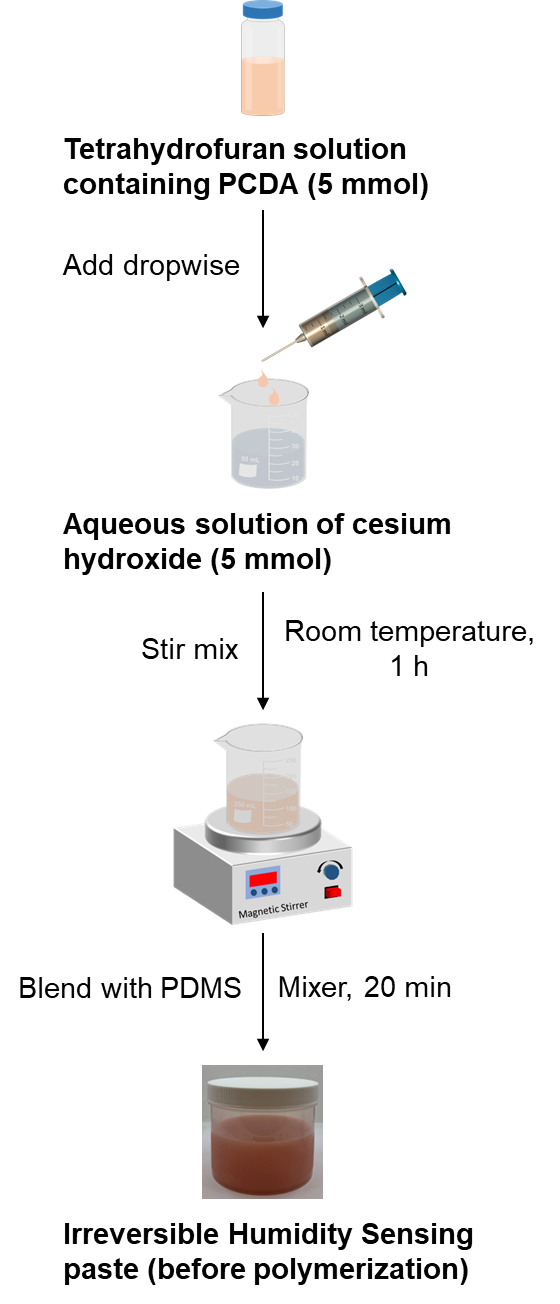
**

**Fig. S9.** Step-by-step preparation process of the irreversible humidity sensing paste, including precursor mixing, solvent addition, magnetic stirring, and the final homogeneous ink formulation.

**
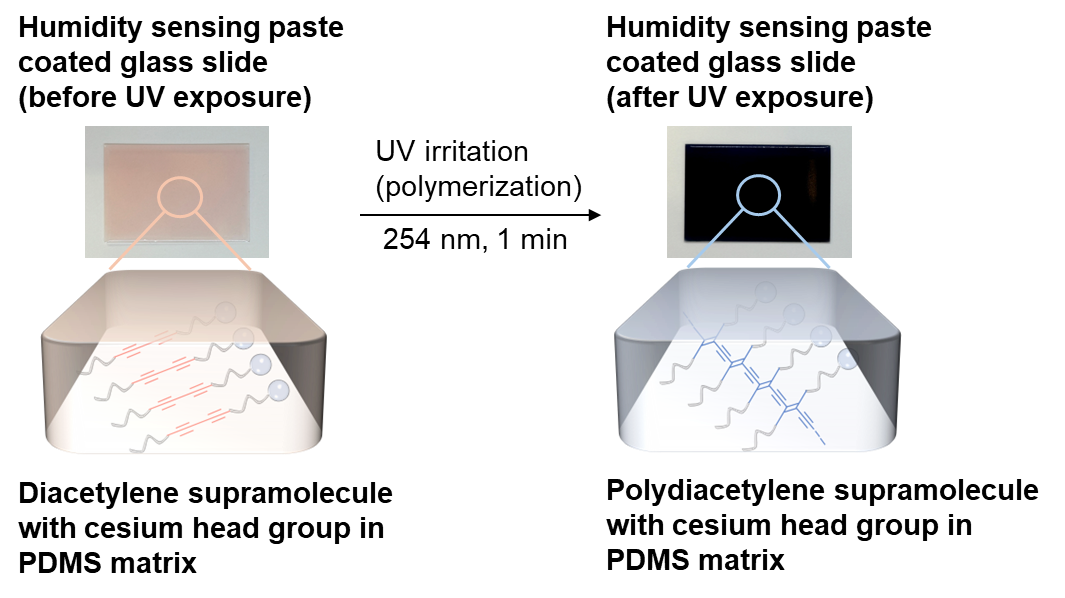
**

**Fig. S10.** Schematic illustration of the polymerization process of the humidity sensor before and after UV exposure, showing the structural transition from dispersed monomers to crosslinked polymer chains.

**
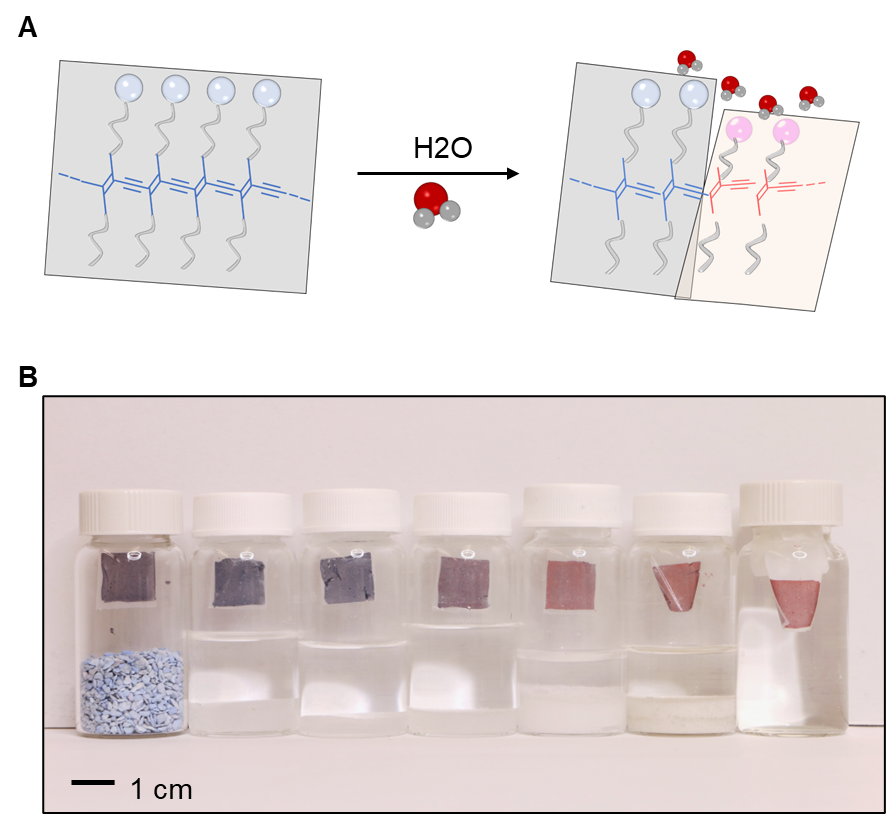
**

**Fig. S11.** Humidity-induced color change of the polydiacetylene-based humidity sensor. (A) Schematic of the molecular response of the polydiacetylene supramolecule with a cesium head group upon exposure to water molecules, showing a color transition triggered by humidity. (B) Photographic sequence showing the color transition of the humidity sensor from blue to red in response to increasing humidity levels (left to right).

**
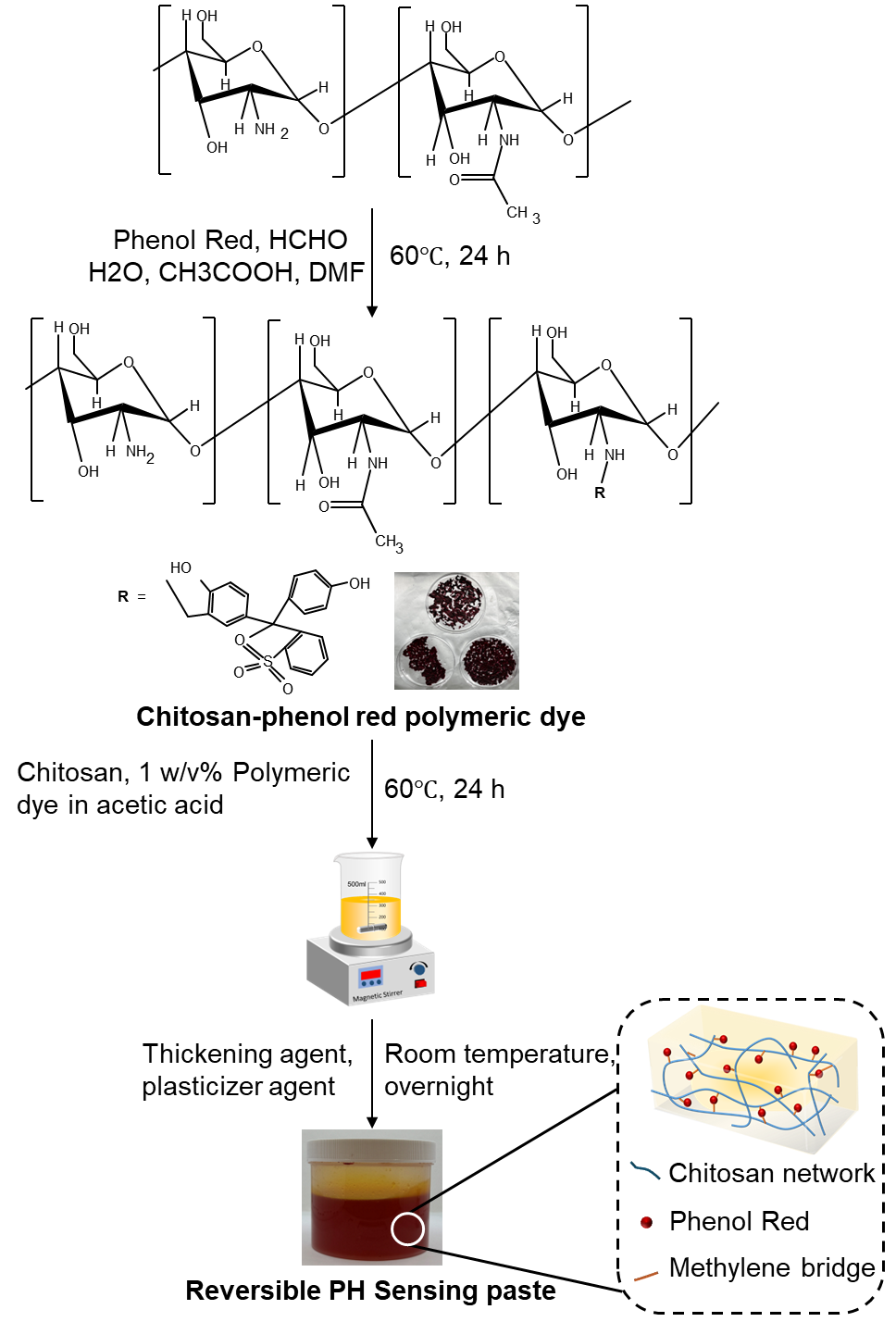
**

**Fig. S12.** Synthesis process of the chitosan–phenol red polymeric dye for reversible pH sensing. The schematic illustrates the Mannich reaction steps, including reagent mixing, dye incorporation, and final polymer formation. The resulting product enables stable integration of pH-responsive dye into the sensor matrix.

**
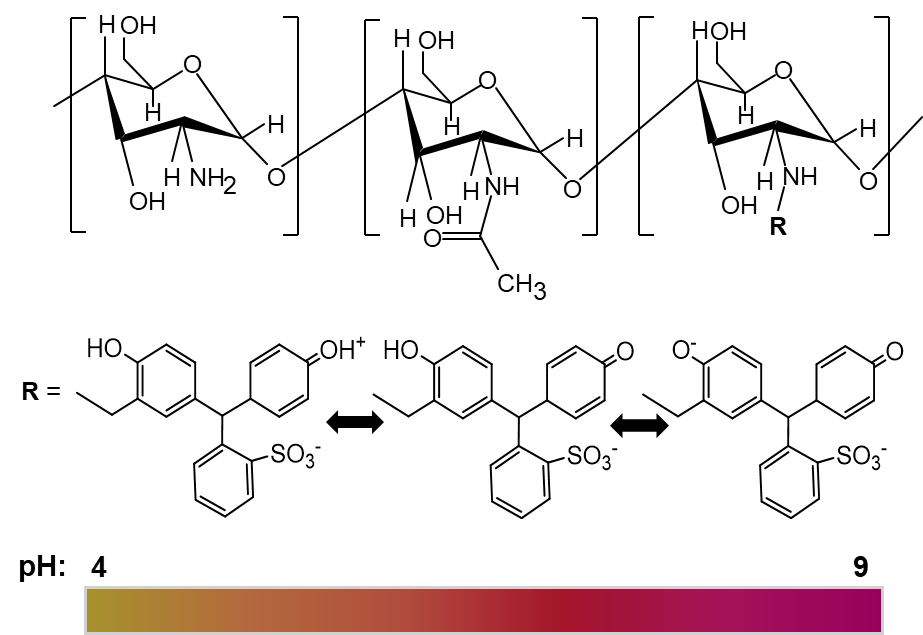
**

**Fig. S13.** Molecular structure and pH-responsive behavior of the chitosan–phenol red polymeric dye. The color gradient illustrates the reversible color transition across the physiological pH range, enabling visual detection of pH changes.

**
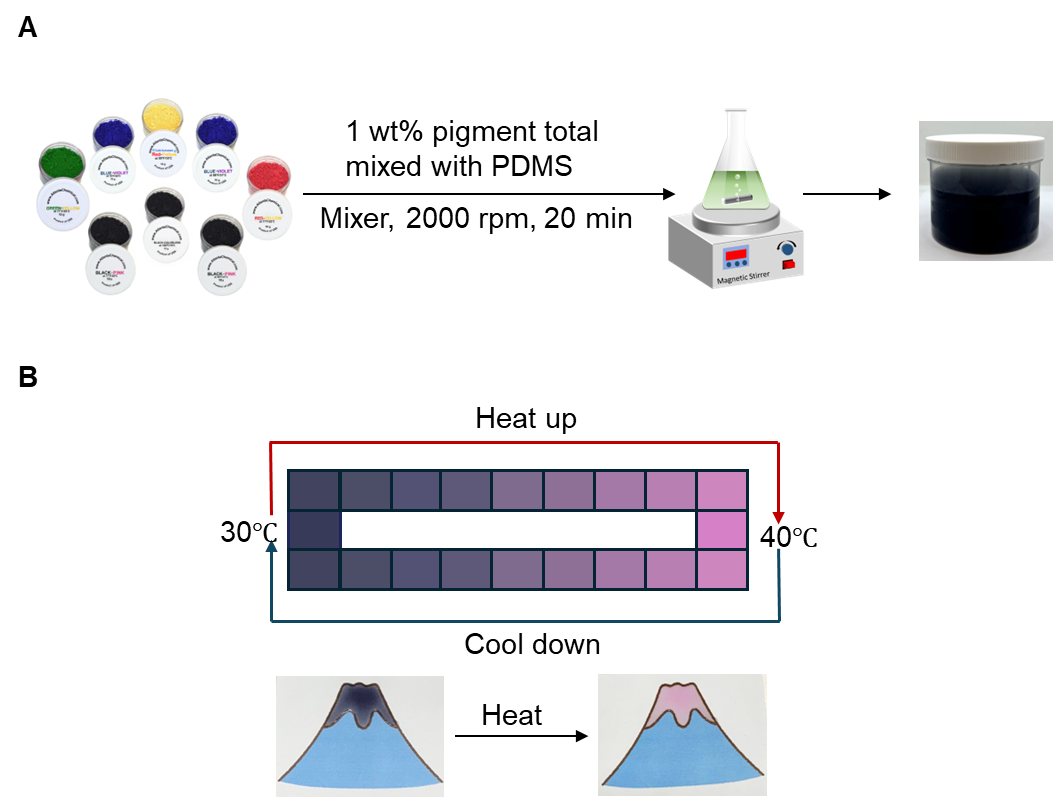
**

**Fig. S14.** Preparation and temperature response of thermochromic sensor. (A) Schematic illustration of the preparation process for the thermochromic sensing ink using thermochromic powder and a PDMS matrix. (B) Reversible color transition of the thermochromic sensor in response to temperature changes. The inset shows a snow mountain design demonstrating the sensor's color shift from 30 °C to 40 °C, highlighting its suitability for reversible temperature monitoring.

**
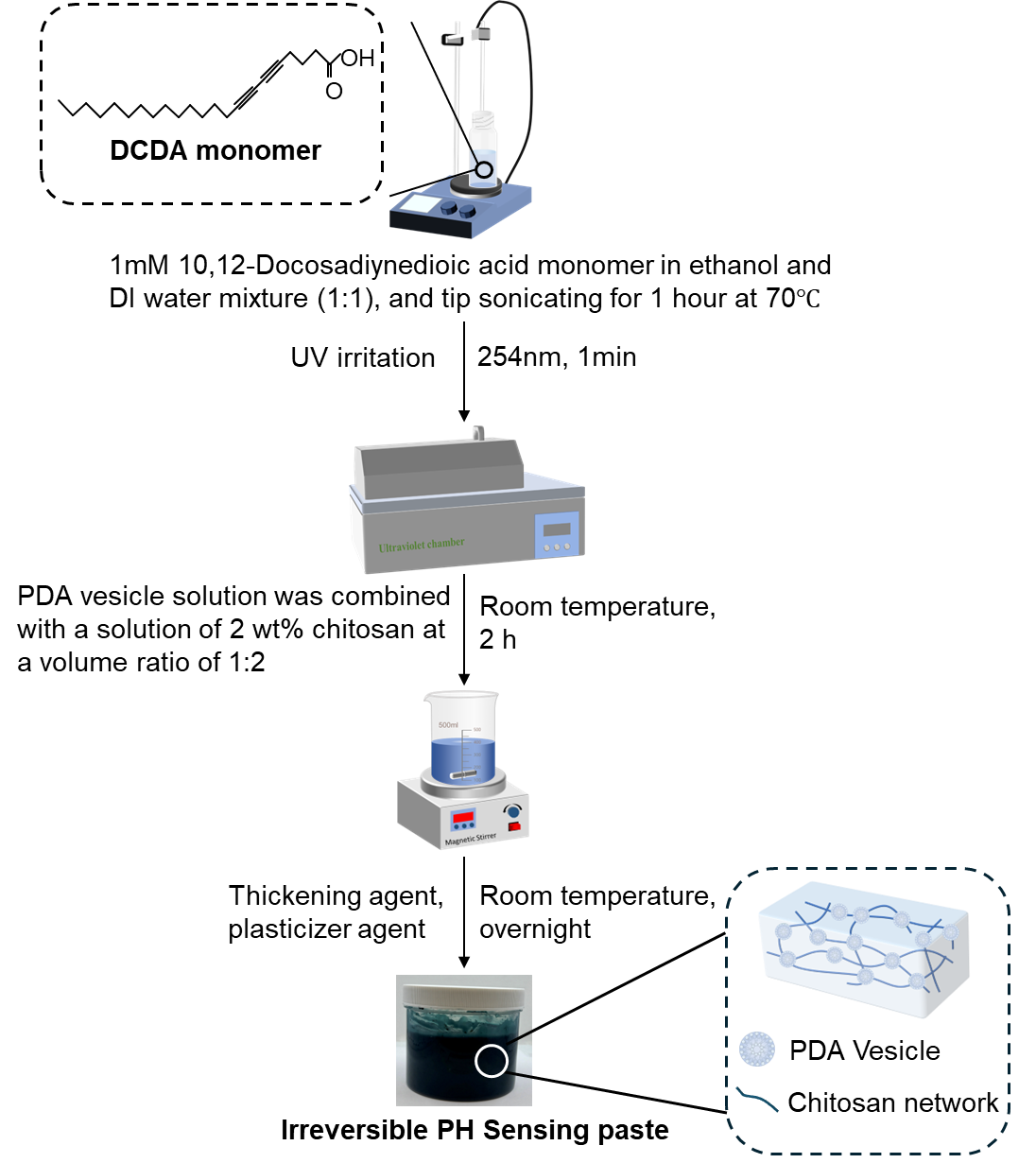
**

**Fig. S15.** Preparation process of the irreversible pH sensing paste using polydiacetylene (PDA) vesicles embedded in a chitosan matrix. The schematic illustrates vesicle formation, UV-induced polymerization, and integration into the chitosan solution to form a stable pH-responsive ink formulation.

**
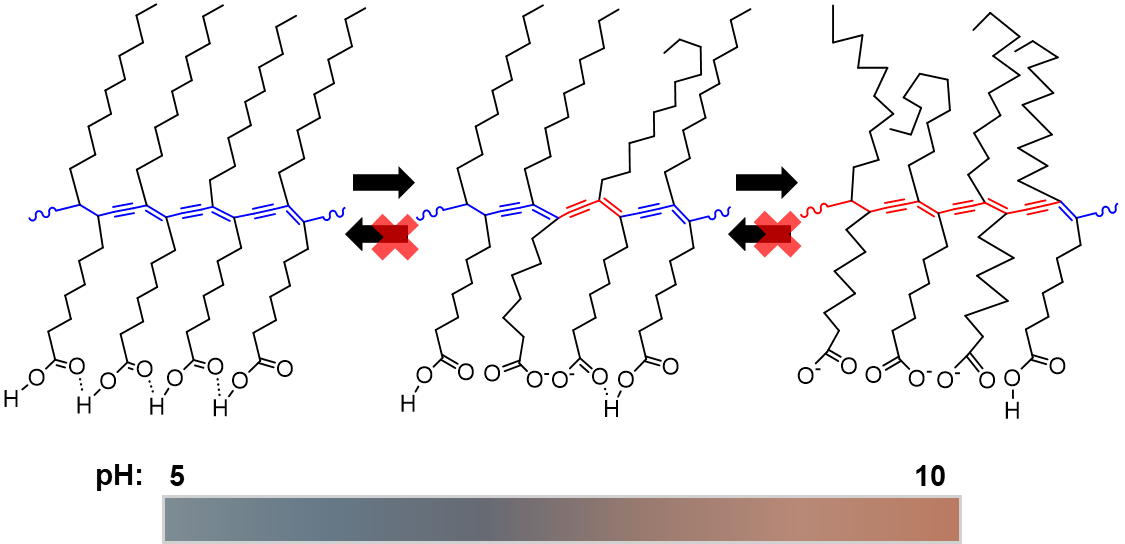
**

**Fig. S16.** Schematic illustration of the colorimetric transition mechanism in the irreversible pH sensor based on PDA vesicles. The blue-to-red color change results from structural disruption of the conjugated backbone in response to alkaline pH, leading to irreversible chromatic shift. The color bar indicates the corresponding visual transition across the pH gradient.

**
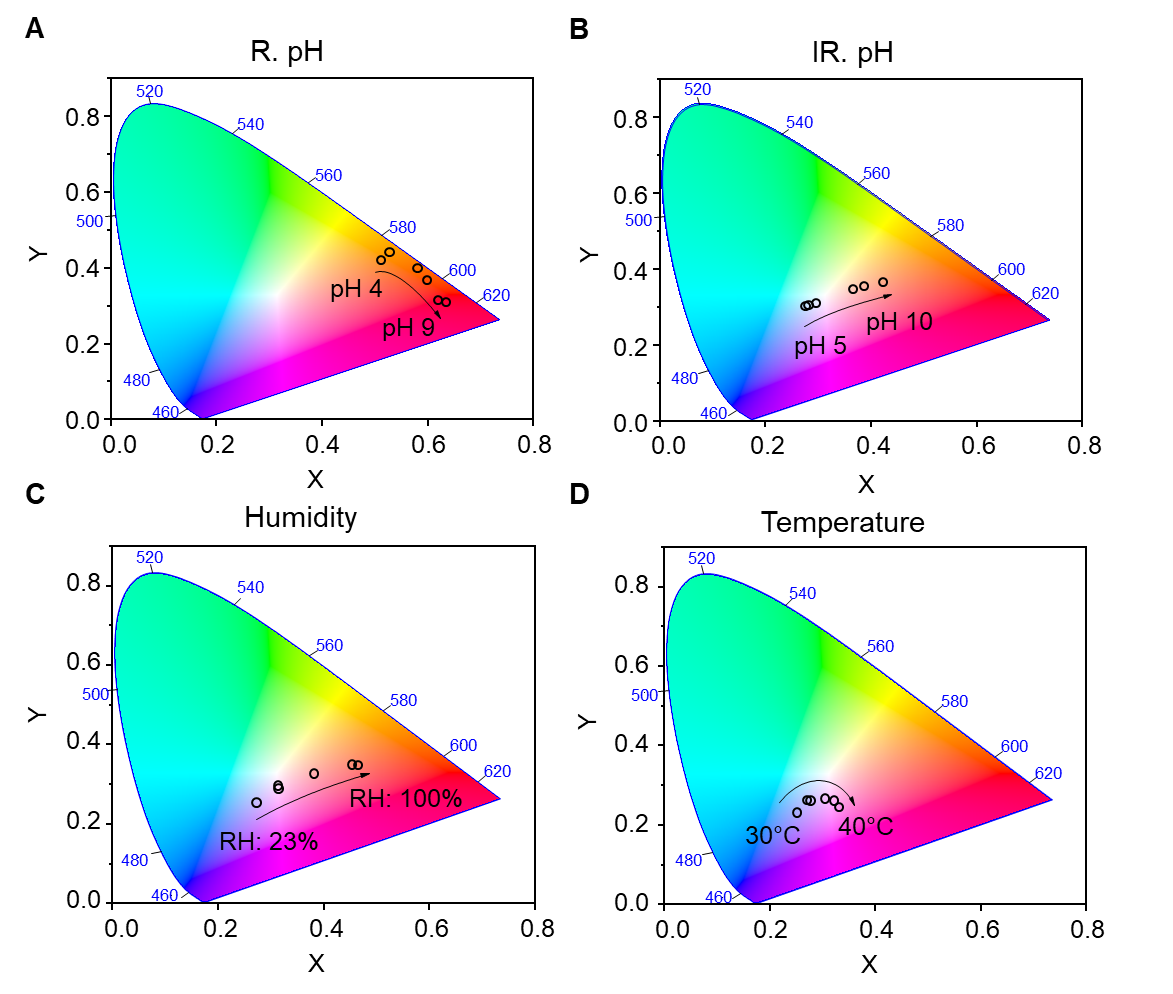
**

**Fig. S17.** CIE 1931 chromaticity diagrams confirming the visible color shifts for (A) reversible pH sensor, (B) irreversible pH sensor, (C) humidity sensor, and (D) temperature sensor. The plots demonstrate distinct trajectory changes in chromaticity coordinates corresponding to each sensor’s responsive range.


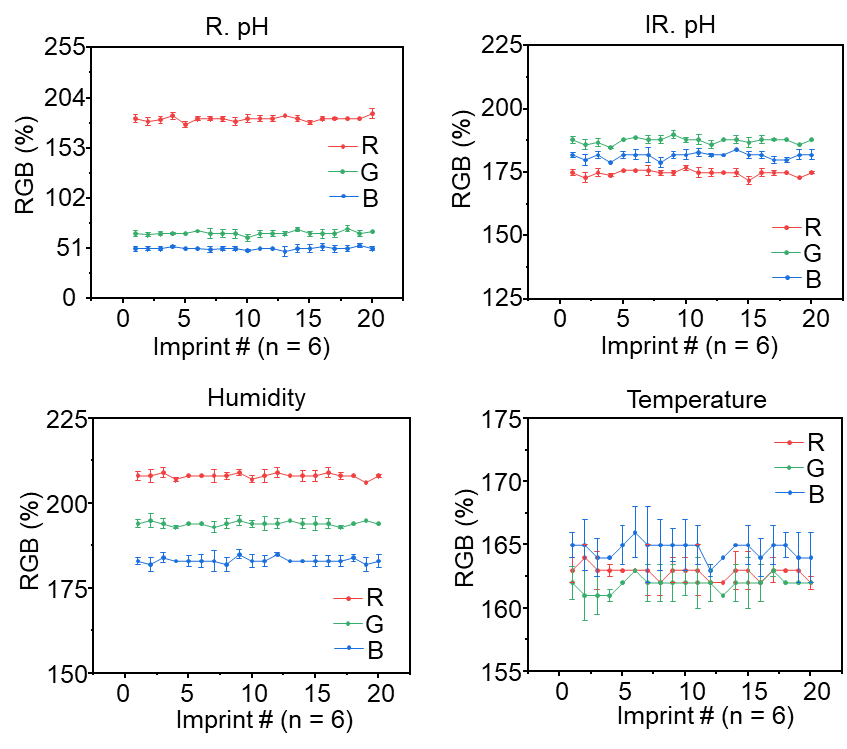


**Fig. S18.** RGB signal consistency across roll-to-roll imprints demonstrating high reproducibility of printed sensors. (Each data point covers 6 samples randomly selected from the same imprint).


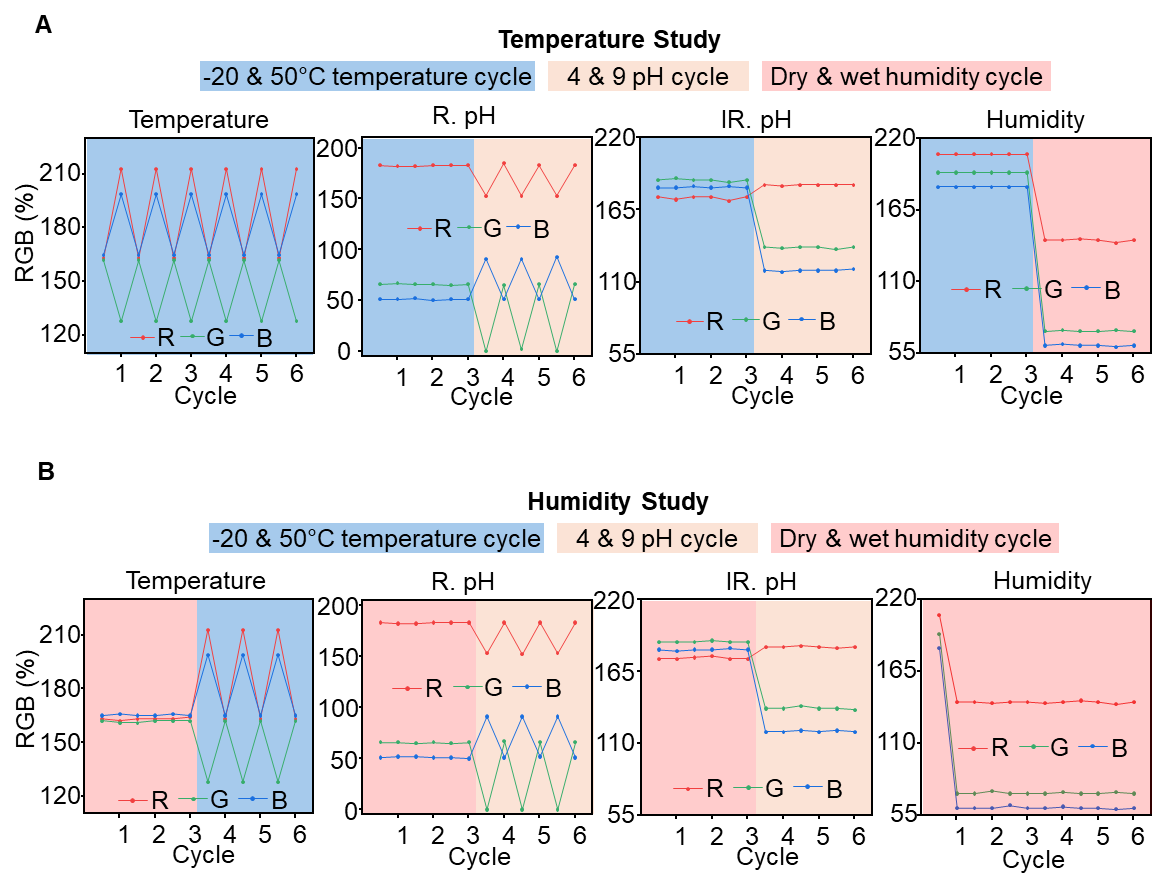


**Fig. S19.** Environmental stability of sensors. (A) Sensor stability under temperature cycles. (B) Sensor stability under humidity cycles.


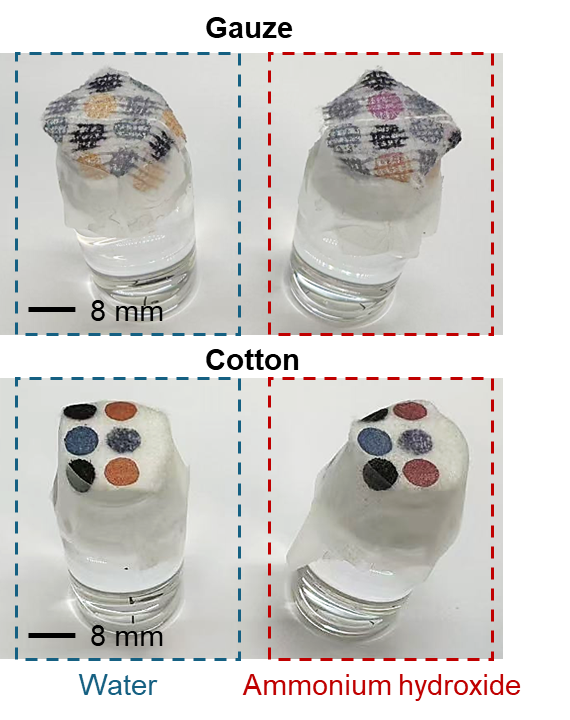


**Fig. S20.** Evaluation of the breathability of the smart wound dressing. Visual comparison of colorimetric pH sensor response under sealed glass vials containing water (left) and ammonium hydroxide (right), demonstrating gas permeability of the dressing that allows vapor diffusion and triggers sensor activation.


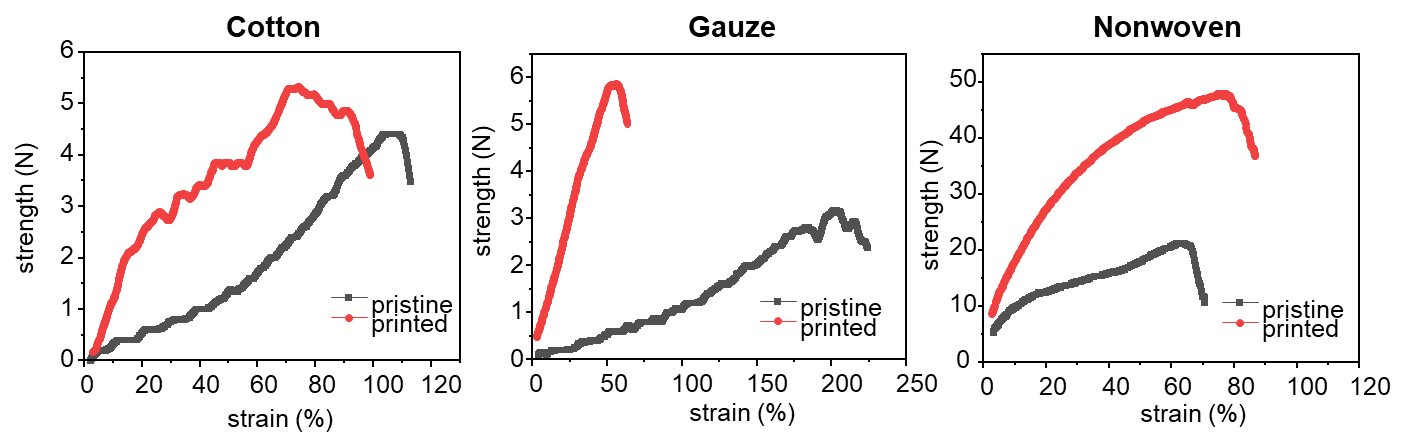


**Fig. S21.** Mechanical stretchability of the smart wound dressing. Stress–strain curves comparing the mechanical behavior of pristine and sensor-integrated dressings under tensile loading, indicating that sensor integration preserves comparable flexibility and stretchability to the original substrate.

**
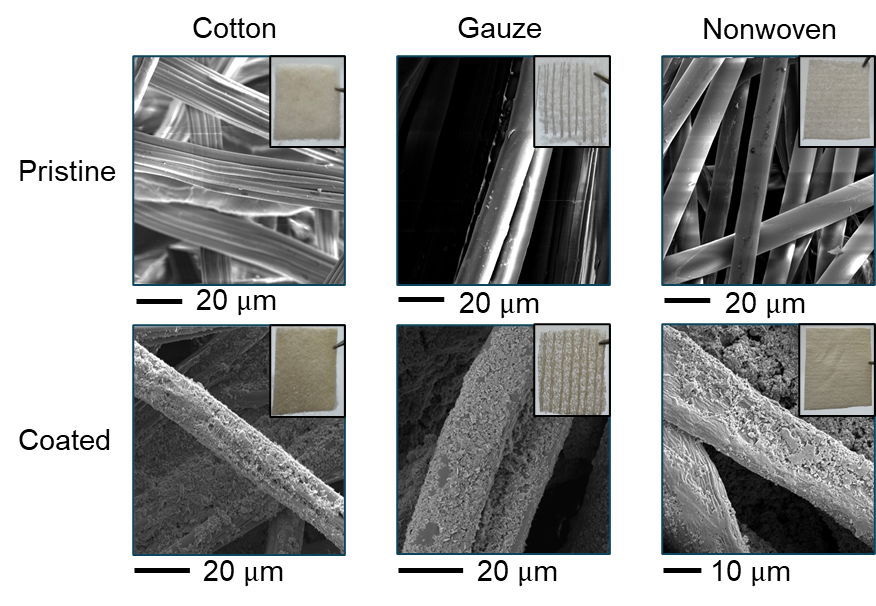
**

**Fig. S22.** SEM of pristine and antifouling material coated fabric (cotton, gauze, nonwoven).

**
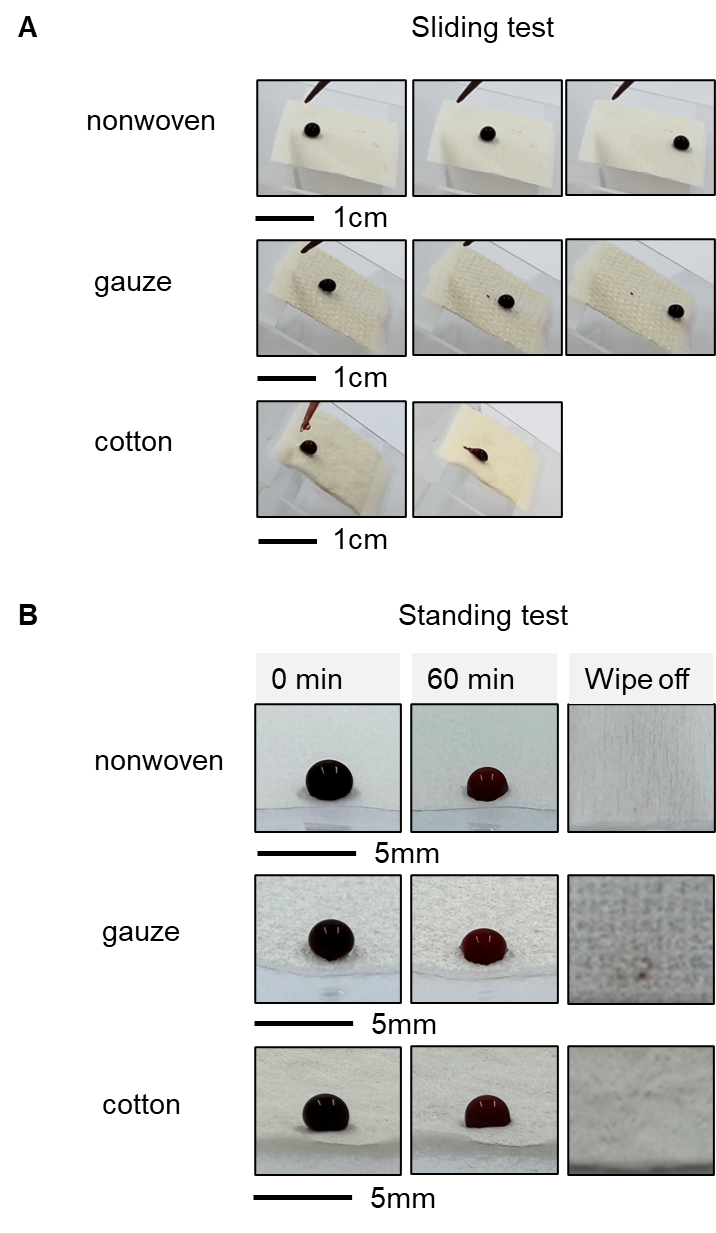
**

**Fig. S23.** (A) Blood sliding behavior on nonwoven, gauze, and cotton dressings coated with the antifouling layer, shown from left to right in chronological order. The cotton dressing exhibits slight blood retention due to the irregular surface texture of cotton fibers. (B) Blood adhesion test showing images at 0 minutes, after 60 minutes, and after wiping, illustrating minimal staining and strong repellency on coated dressings.

**
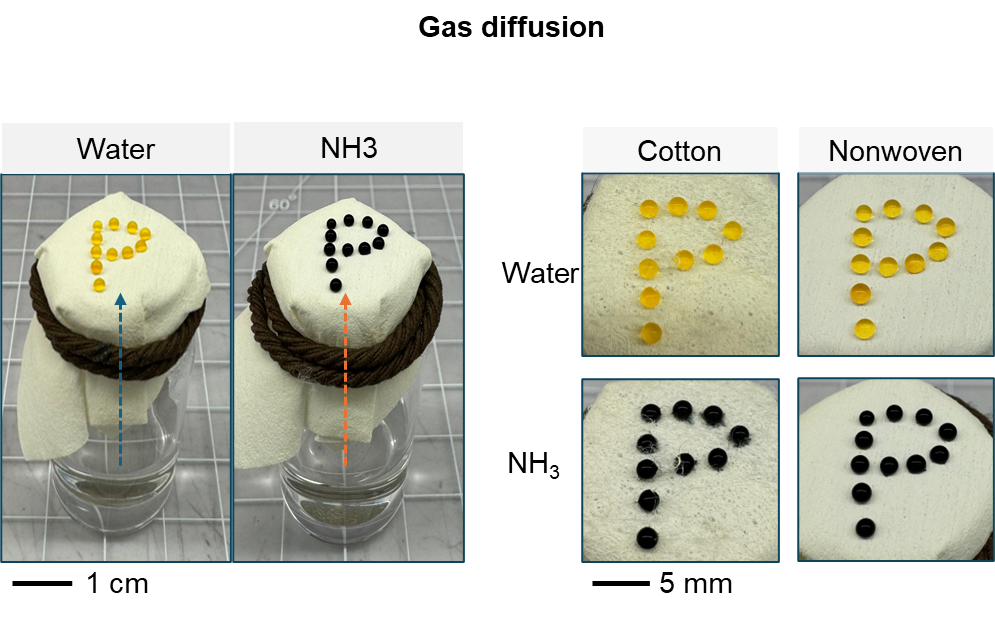
**

**Fig. S24.** Gas permeability assessment of the antifouling-coated dressing. Bromothymol blue (BTB) droplets exhibit a clear colorimetric response to ammonia (NH₃) vapor diffusion, indicating that the antifouling coating retains breathability on both cotton and nonwoven substrates. Gauze dressing was not tested due to its inherently high porosity.

**
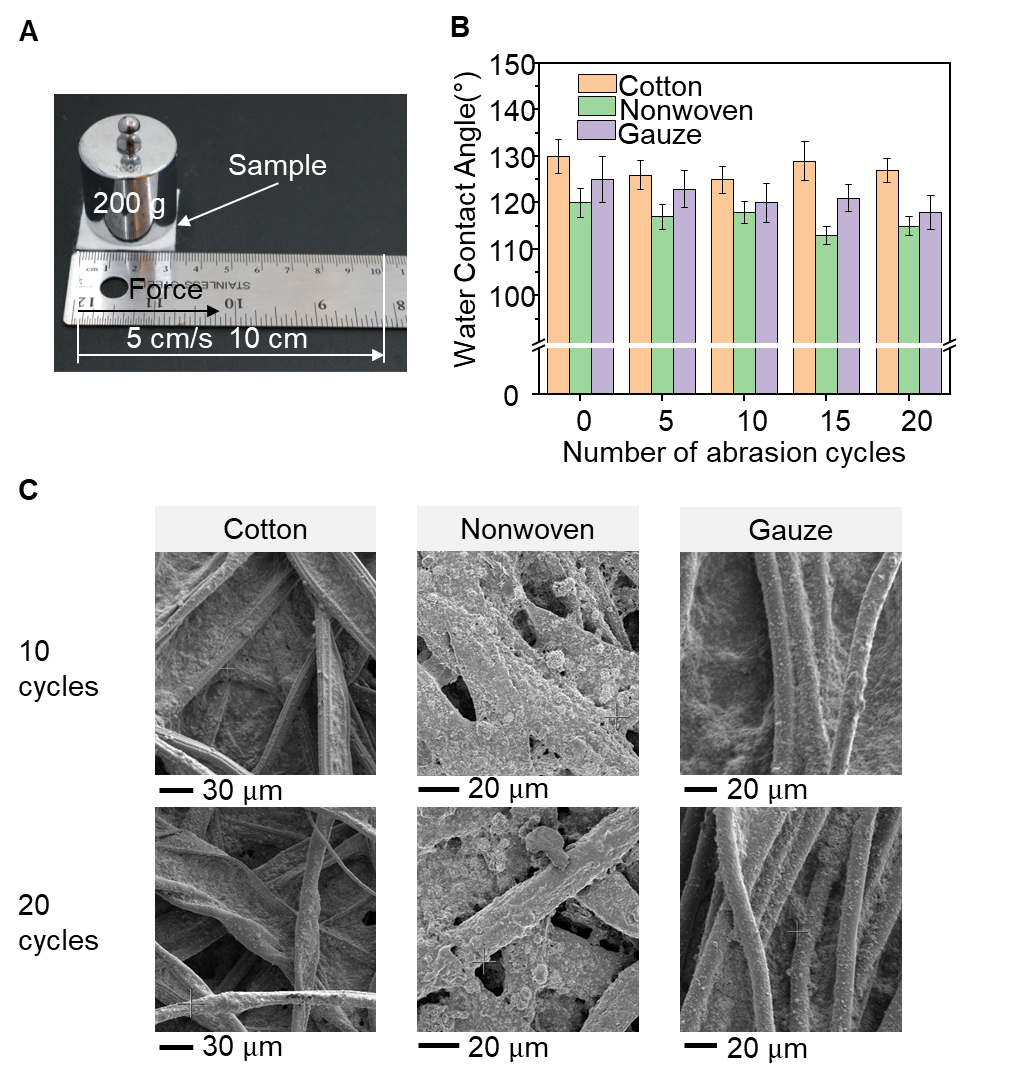
**

**Fig. S25.** Mechanical durability of antifouling coatings on different dressing materials. (A) Setup for abrasion test with a sample subjected to repeated abrasion cycles under a 200 g load at 5 cm/s. (B) Water contact angle measurements for cotton, nonwoven, and gauze dressings over 20 abrasion cycles, indicating retained hydrophobicity. (C) SEM images of cotton, nonwoven, and gauze samples after 10 and 20 abrasion cycles.

**
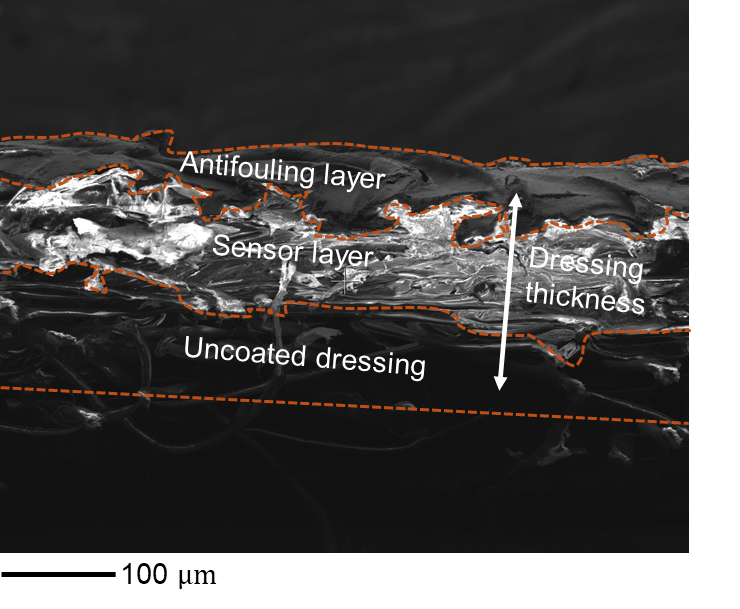
**

**Fig. S26.** Cross-sectional SEM image of the smart wound dressing fabricated on a nonwoven substrate, showing distinct layers including the uncoated dressing base, sensor layer, and antifouling top layer.

**
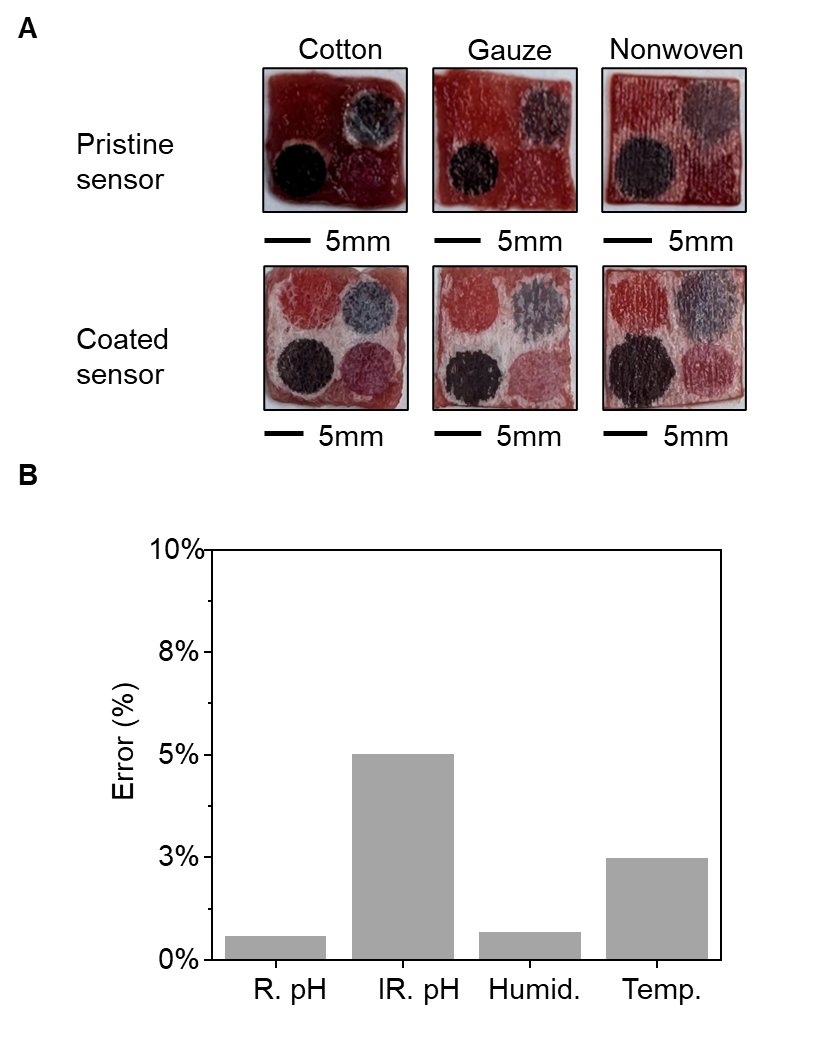
**

**Fig. S27.** Color calibration of the smart wound sensor using artificial wound fluid (AWF) at pH 9 and 40 °C. (A) Images of pristine and antifouling-coated sensor patches after 30 minutes of soaking in AWF at room temperature, demonstrating the effectiveness of the antifouling layer. (B) Absolute percentage error (Error) of color quantification result.


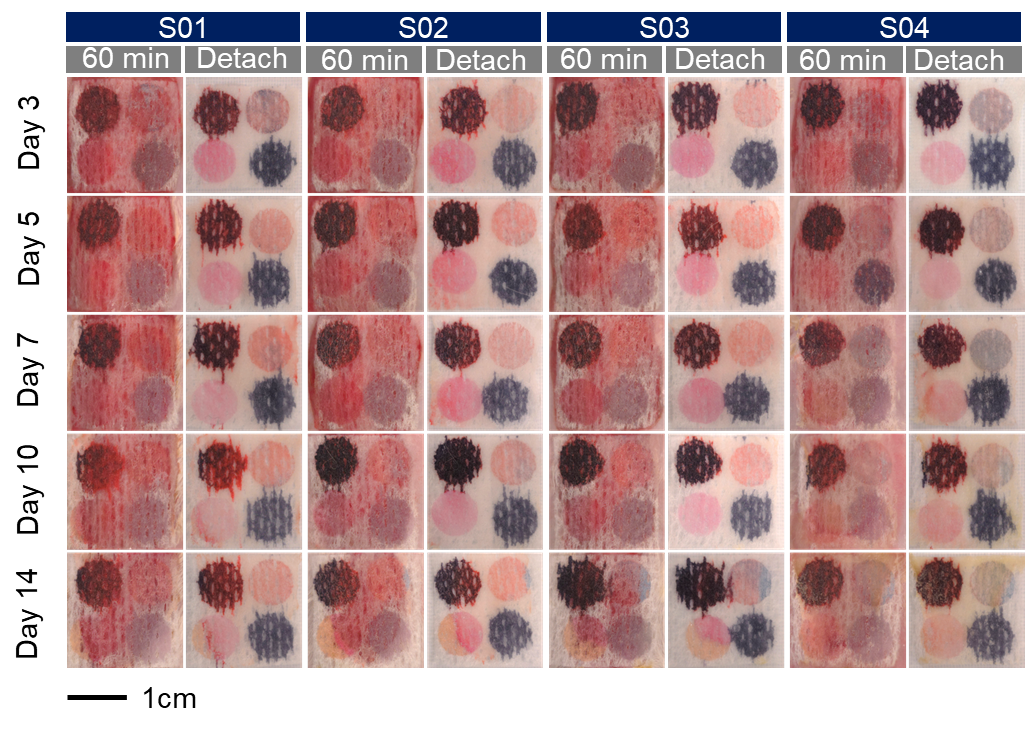


**Fig. S28.** Longitudinal monitoring of sensor color changes on non-infected wounds over 14 days. Images of smart dressing sensors on non-infected wounds (S01 to S04) at Days 3, 5, 7, 10, and 14, with observations recorded at 60 minutes post-application (temperature) and upon detachment.


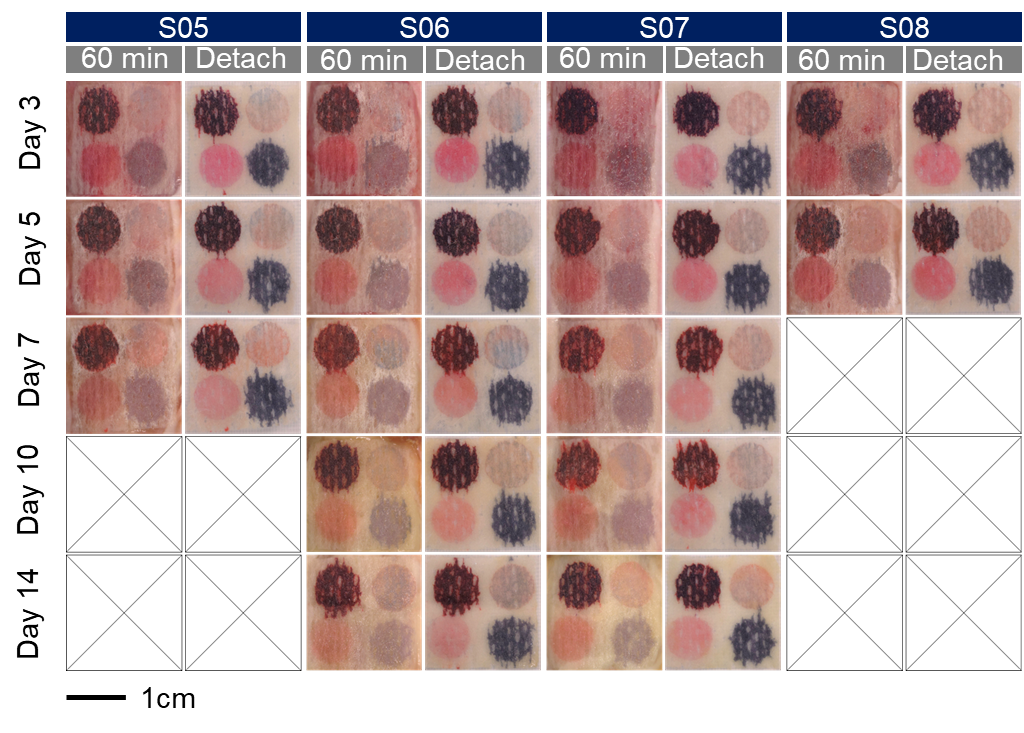


**Fig. S29.** Longitudinal monitoring of sensor color changes on infected wounds over 14 days. Images of smart dressing sensors on infected wounds (S05 to S08) at Days 3, 5, 7, 10, and 14, with observations recorded at 60 minutes post-application (temperature) and upon detachment (S05 and S08 mouse were sacrificed on Day 10 and Day 7 respectively due to severe infection).


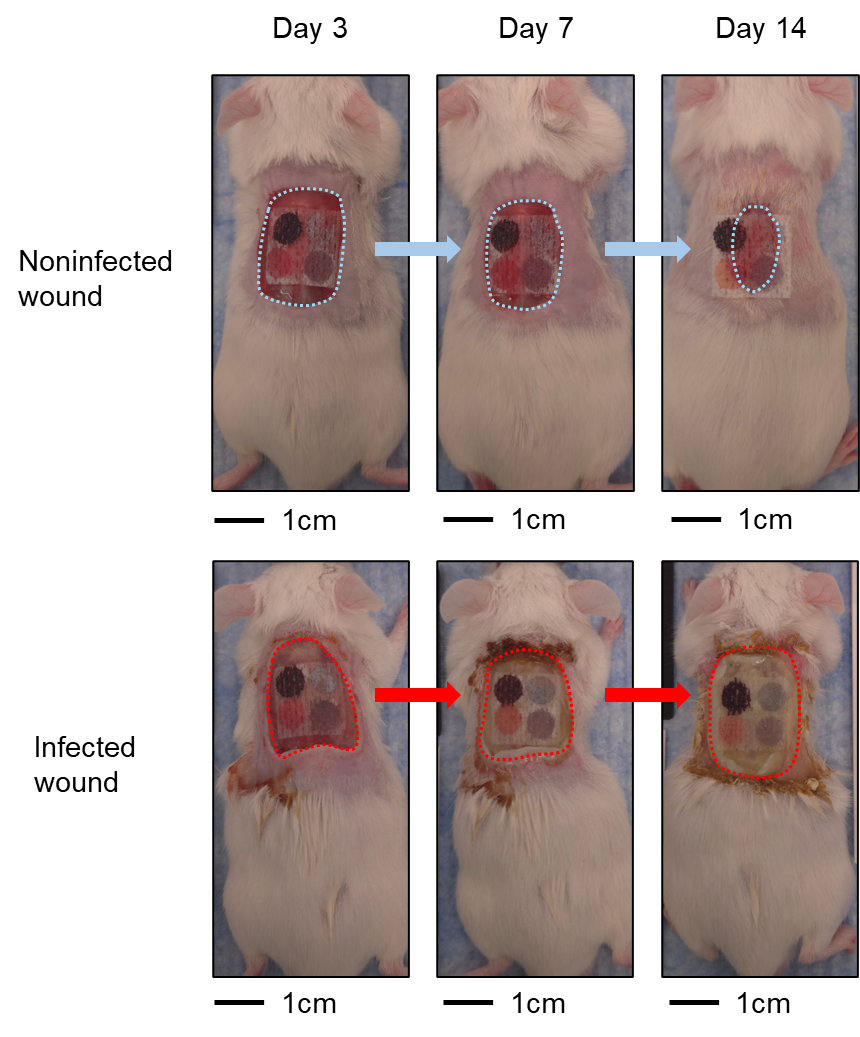


**Fig. S30.** Photographs showing wound size progression in non-infected and infected wounds on Days 3, 7, and 14. Blue dotted outlines represent non-infected wounds, while red outlines indicate infected wounds, illustrating delayed healing and persistent inflammation in the infected group.

**
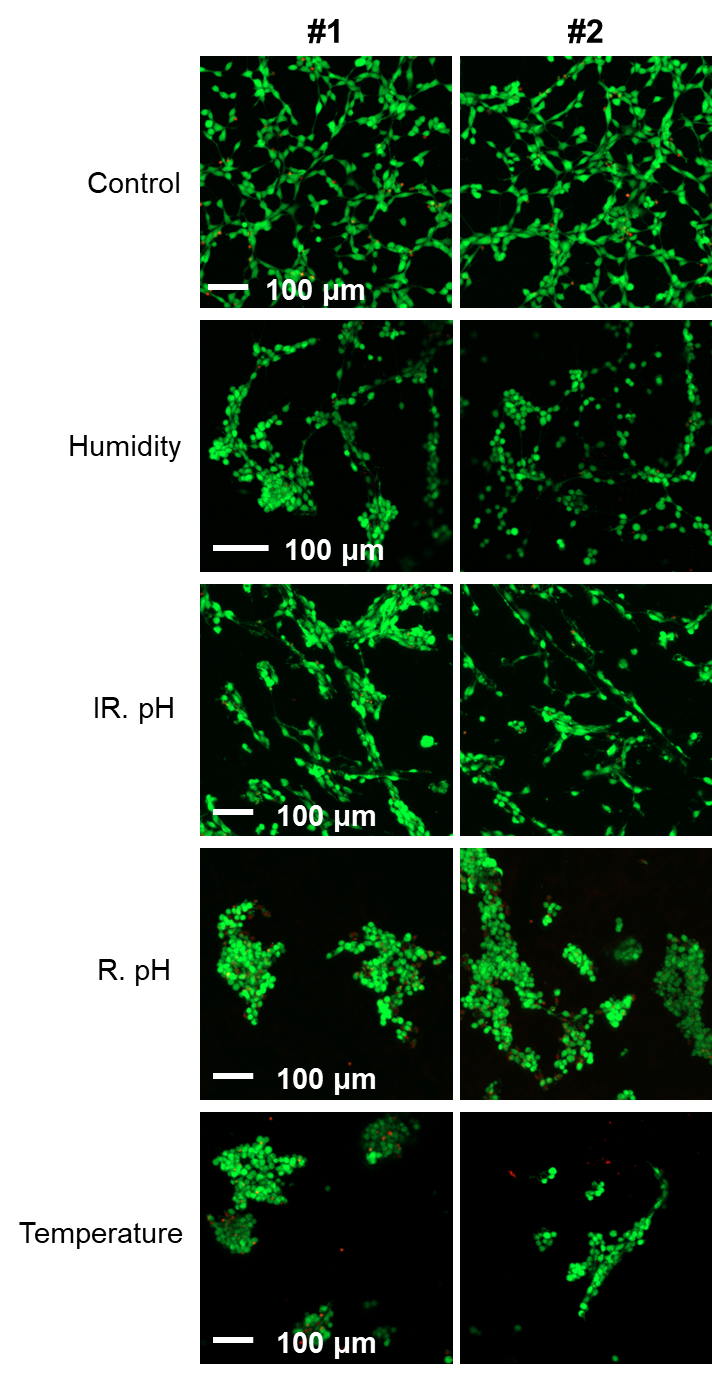
**

**Fig. S31.** Fluorescence images of cell viability for control (culture dish) and sensor-coated dressing samples, including humidity, irreversible pH (IR pH), reversible pH (R pH), and temperature sensors. Images were taken from two independent culture wells (#1 and #2) on the same day to assess reproducibility. Green fluorescence indicates viable cells (Live/Dead staining).

**
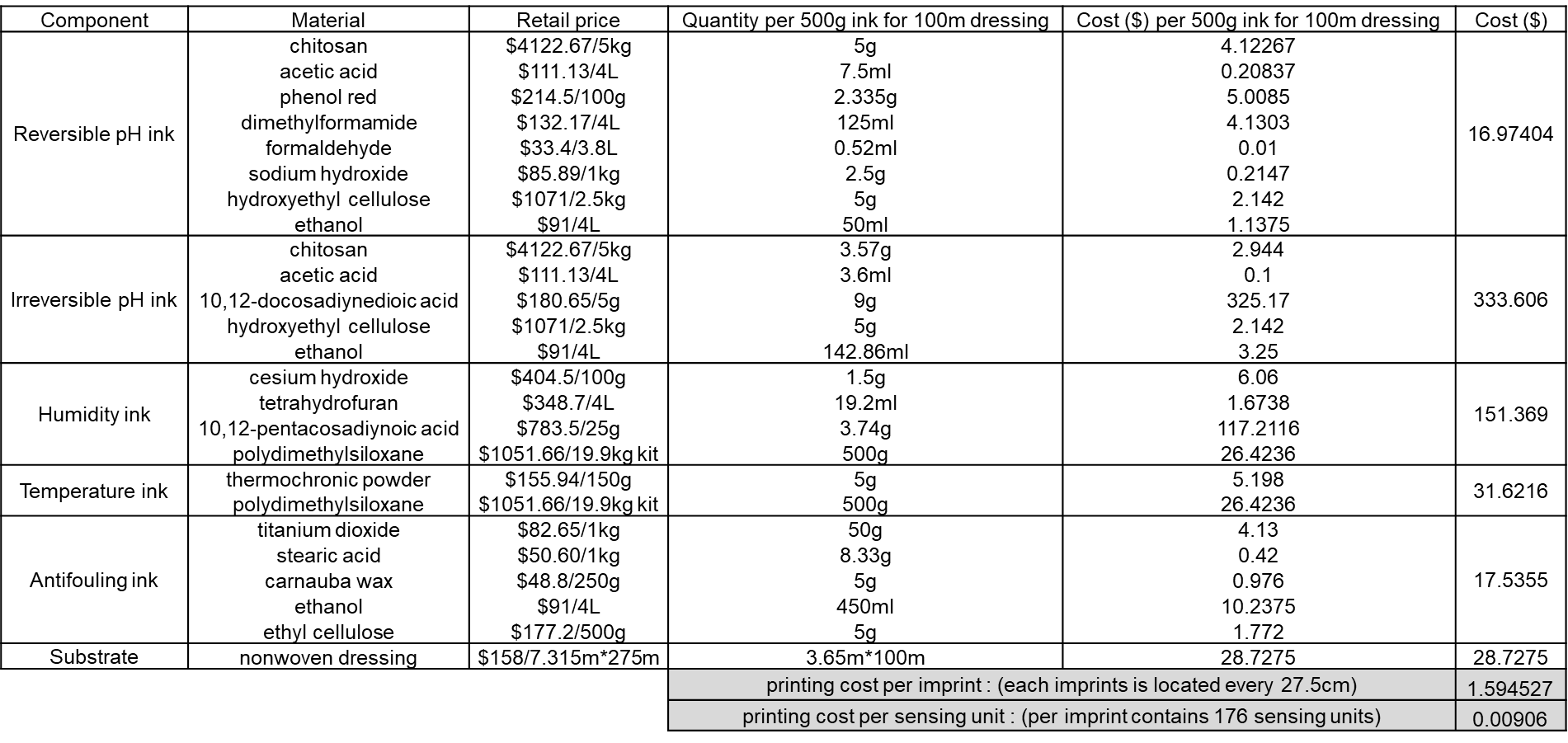
**

**Table S1.** Estimated material costs of sensing and functional inks, individual imprints, and per-unit sensors in the smart wound dressing based on retail prices.

| **Wound marker** | **Clinical significance** | **Healthy wound** | **Infected wound** | **Ischemic wound** | **Ref.** |
| --- | --- | --- | --- | --- | --- |
| pH | Wound pH is closely linked to wound healing process, bacterial activity, and cellular function. | Slightly acidic to neutral. (pH 6.5 to 8.5) | Increased pH, alkaline shift. (pH 7.2 to 8.9) | Tissue pH drops due to anaerobic metabolism, producing lactic acid. | ^[1-3]^ |
| Temperature | Temperature indicates blood flow, inflammation, and metabolic activity in wound tissue. | 31 to 35°C | Increased temperature relative to healthy skin level. (35 to 38°C) | Decreases due to reduced blood flow, lower than surrounding tissue. | ^[4-7]^ |
| Humidity | Moisture balance is essential for optimal healing; excessive or insufficient moisture impairs repair. | moist, non-macerating | Increased exudate accumulation and high moisture, maceration | Ischemic wounds exhibit lower moisture levels due to reduced perfusion and fluid production | ^[8-11]^ |

**Table S2.** Clinical significance of the selected biomarkers in healthy, infected and ischemic wounds.

| **Relative Humidity (RH) Source** | **RH (%)** | **Ref.** |
| --- | --- | --- |
| Desiccant | 0 | ^[12]^ |
| Saturated potassium hydroxide -KOH | 9 | ^[13]^ |
| Saturated potassium acetate -CH_3_CO_2_K | 23 | ^[14]^ |
| Saturated magnesium nitrate -Mg (NO_3_)_2_ | 54 | ^[14]^ |
| Saturated Sodium Chloride ‐NaCl | 75 | ^[12]^ |
| Saturated potassium sulphate -K_2_SO_4_ | 98 | ^[12]^ |
| Water | 100 | ^[12]^ |

**Table S3.** Standard saturated salt solutions and corresponding static relative humidity environments at room temperature.

**Movie S1.**

R2R fabrication process.

**Movie S2.**

Smart wound dressing: on body demonstration.

**Movie S3.**

Water drawing on PDA and PDMS/PDA coated glass slides showing different reaction speed.

**Movie S4.**

Reversible pH sensing.

**Movie S5.**

Reversible response of temperature sensor.

**Movie S6.**

Irreversible pH sensing.

**References**

[1] R. B. Louisa, N. M. Charne, J. S. Richard, M. B. M. Andrea, S. Geoff, M. William, *Wound Pract. Res.* **2017**, 25.

[2] P. Sim, X. L. Strudwick, Y. Song, A. J. Cowin, S. Garg, *Int. J. Mol. Sci.* **2022**, 23, 13655.

[3] S. Chien, B. J. Wilhelmi, *J Vis Exp* **2012**, e3341.

[4] X. T. Zheng, Z. Yang, L. Sutarlie, M. Thangaveloo, Y. Yu, N. A. B. M. Salleh, J. S. Chin, Z. Xiong, D. L. Becker, X. J. Loh, B. C. K. Tee, X. Su, *Sci. Adv.* **2023**, 9, eadg6670.

[5] R. He, H. Liu, T. Fang, Y. Niu, H. Zhang, F. Han, B. Gao, F. Li, F. Xu, *Adv. Sci.* **2021**, 8, 2103030.

[6] M. Fierheller, R. G. Sibbald, *Adv. Skin Wound Care.* **2010**, 23.

[7] G. Power, Z. Moore, T. O'Connor, *J. Wound Care* **2017**, 26, 381.

[8] D. Okan, K. Woo, E. A. Ayello, G. Sibbald, *Adv. Skin Wound Care.* **2007**, 20, 39.

[9] S. Chien, B. J. Wilhelmi, *A Simplified Technique for Producing an Ischemic Wound Model*, Vol. 63, 1940-087X, **2012**.

[10] J. G. Powers, C. Higham, K. Broussard, T. J. Phillips, *J. Am. Acad. Dermatol.* **2016**, 74, 607.

[11] J. P. E. Junker, R. A. Kamel, E. J. Caterson, E. Eriksson, *Adv. Wound Care.* **2013**, 2, 348.

[12] M. Momtaz, J. Chen, *ACS Appl. Mater. Interfaces.* **2020**, 12, 54104.

[13] M. E. Solomon, *Bull. Entom. Res., Lond.* **1951**, 42, 543.

[14] L. Greenspan, *J. Res. Natl. Bur.* **1977**, 81, 89.
